# Early Evidence for Northern Salmonid Fisheries Discovered using Novel Mineral Proxies

**DOI:** 10.1038/s41598-018-36133-5

**Published:** 2019-01-16

**Authors:** D. H. Butler, S. Koivisto, V. Brumfeld, R. Shahack-Gross

**Affiliations:** 1 Laboratory for Sedimentary Archaeology, Department of Maritime Civilizations, University of Haifa, 199 Abba Khoushy Ave, Haifa, 3498838 Israel; 2 Department of Cultures/Archaeology, University of Helsinki, Unioninkatu 38 F, Helsinki, FI-00014 Finland; 3 Electron Microscopy Unit, The Weizmann Institute of Science, Rehovot, 234 Herzl St, Rehovot, 76100 Israel

## Abstract

Salmonid resources currently foster socioeconomic prosperity in several nations, yet their importance to many ancient circumpolar societies is poorly understood due to insufficient fish bone preservation at archaeological sites. As a result, there are serious gaps in our knowledge concerning the antiquity of northern salmonid fisheries and their impacts on shaping biodiversity, hunter-gatherer adaptations, and human-ecological networks. The interdisciplinary study presented here demonstrates that calcium-magnesium phosphate minerals formed in burned salmonid bones can preserve at ancient northern sites, thus informing on the early utilization of these resources despite the absence of morphologically classifiable bones. The minerals whitlockite and beta magnesium tricalcium phosphate were identified in rare morphologically classifiable Atlantic salmonid bones from three Mid-Holocene sites in Finland. Large amounts of beta magnesium tricalcium phosphate were also experimentally formed by burning modern Atlantic salmonid and brown trout bones. Our results demonstrate the value of these minerals as proxies for ancient northern salmonid fishing. Specifically, the whitlockite mineral was discovered in hearth sediments from the 5,600 year old Yli-Ii Kierikinkangas site on the Iijoki River in northern Finland. Our fine sieving and mineralogical analyses of these sediments, along with zooarchaeological identification of recovered bone fragments, have confirmed for the first time that the people living at this village did incorporate salmonids into their economies, thus providing new evidence for early estuary/riverine fisheries in northern Finland.

## Introduction

**Figure 1:**
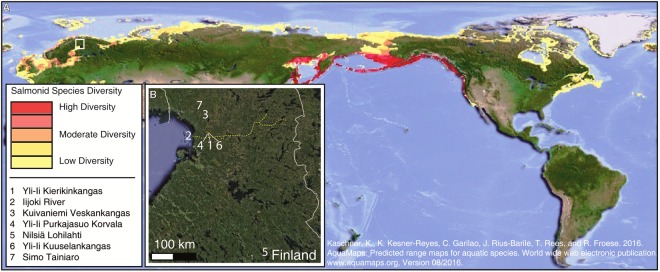
Global salmonid distribution and the study region. (**A**) Map illustrating the global distribution of salmonids. Note the wide-spread availability of salmonids in high, low, and sub Arctic regions. (**B**) Map of Finland marking the locations of the Yli-Ii Kierikinkangas site (1) and the Iijoki River (2), where much archaeological research has focused on searching for indicators of salmonid utilization. Three archaeological sites found to contain burned Atlantic salmonid bone represent half of the examples for the entire archaeology of Finland (3, 4, 5). Other sites mentioned in the text are marked as well (6, 7).

The coalescence of ecological, anthropological, and archaeological research has provided comprehensive insight into the relationships between fish resources, aquatic environments, and human activities at multiple geographic and temporal scales^1–3^. Salmonids in specific are widely available, and their influence on human niche construction, social structuring, and landscape politics is well-documented in places such as Japan and the northwest coast of North America^4,5^. Salmonids are also abundant throughout the high, low, and sub Arctic, yet the depth of their ancient use across these regions is poorly understood because of the rarity of well-preserved fish remains at archaeological sites (Fig. 1A and Supplementary Information 1A,B). This scarcity is thought to result from the loss of fish bones to animal scavenging, the return of remains to water, ceremonial cremation, disposal by burning, and low resolution archaeological recovery methods^6–12^. Post-depositional dissolution owing to the prevailing acidic nature of regional soils and sediments is another major reason for the paucity of ancient salmonid bone in the north^7,8^. Salmonid bone quickly decomposes in acidic contexts because of its large organic content, loosely packed collagen bundles, and high porosity^13,14^. All of these processes have hindered the development of broad understandings of the antiquity, geography, and ecology of northern human-salmonid interactions. Addressing this issue, we developed a new mineralogical-proxy-based methodology for identifying the past use of salmonid resources when morphologically classifiable fish bones have not been preserved.

The historic importance of salmonid fisheries in northern North America and Europe has led many archaeologists to speculate that they must have been equally important in prehistory, despite the fact that zooarchaeological and material culture indicators for such fisheries are in short supply (Supplementary Information 1A,B). This report focuses on northwest Finland, where mass‐harvesting of Atlantic salmonid formed the basis of a thriving coastal-river economy far into the 20^th^ century, up until the major rivers were harnessed to produce hydroelectric power^8^. Rivers flowing into the northern end of the Gulf of Bothnia directed hundreds of thousands of Atlantic salmonid into the Baltic Sea every year. At River Kemijoki in south Lapland, for example, several hundred tons of salmonid were harvested seasonally throughout the 17^th^ and 18^th^ centuries^15^. Much less is understood about the use of salmonid resources further back in time^8–12^. Only six morphologically classifiable Atlantic salmonid bones have been recovered from the thousands of sites dating to the (Sub-) Neolithic (typically referred to as non-agricultural Neolithic or pottery Mesolithic, ca. 7,200–5,300 years BP) (Fig. 1B and Supplementary Information 1B)^8^. This absence of evidence is certainly not evidence of absence, yet pinpointing where and when Atlantic salmonid, and other types of fish, were utilized without relying on the recovery of well-preserved bone is a methodological challenge that has not been fully resolved, even on the coarse scale of presence/absence.

DNA profiles, peptide fingerprints, and isotopic signatures can be very useful for determining whether fish derived residues are present at prehistoric sites; yet, these methods are best suited for analyses of preserved collagen^5^^,^^6^. They are of limited use in situations where highly burned bone fragments dominate faunal assemblages, as these specimens lack the collagen needed for the aforementioned analyses. Such is the case in Finland, where the disposal of bone refuse in domestic fires is thought to have been a wide-spread waste management strategy throughout prehistory^11,12^. Burned fish bone fragments, however, may be preserved in the sediments comprising these combustion features. In a recent study we found that rare calcium-magnesium phosphate minerals form in salmonid bones burned at the relatively low temperatures typical of open wood-fuelled hearths (400–600 °C), and we postulated that these minerals have the potential to serve as proxy indicators for ancient salmonid fisheries^16^. The validity of this premise, however, relies on the preservation of burned salmonid bone fragments and associated neoformed minerals in acidic northern sediments.

Here, we use Fourier transform infrared spectroscopy (FTIR), x-ray diffraction (XRD), and micro-computed tomography (µCT) to (1) show that calcium-magnesium phosphate minerals are viable indicators for burned salmonid bone particles in archaeological sediments, and (2) understand the mechanisms facilitating the preservation of these minerals. These aims are achieved through a study of rare Atlantic salmonid bones from three Late Mesolithic/(Sub-) Neolithic sites in Finland, comparing them to bones of other identified game from these sites, studying hearth sediments from a site where classifiable salmonid bones have not been recovered (the 5,600 year old Yli-Ii Kierikinkangas site), and conducting a set of laboratory experiments involving burning and hydrating modern fish bones (Fig. 1B and Supplementary Information 1C). We confirm that calcium-magnesium phosphate minerals are valuable proxies for ancient salmonid fisheries and that hydration is a key mechanism promoting the preservation of these minerals. Our mineralogical-proxy-based approach will open new vistas for tracking dynamics in northern riverine and coastal human-ecologies.

## Results

**Figure 2:**
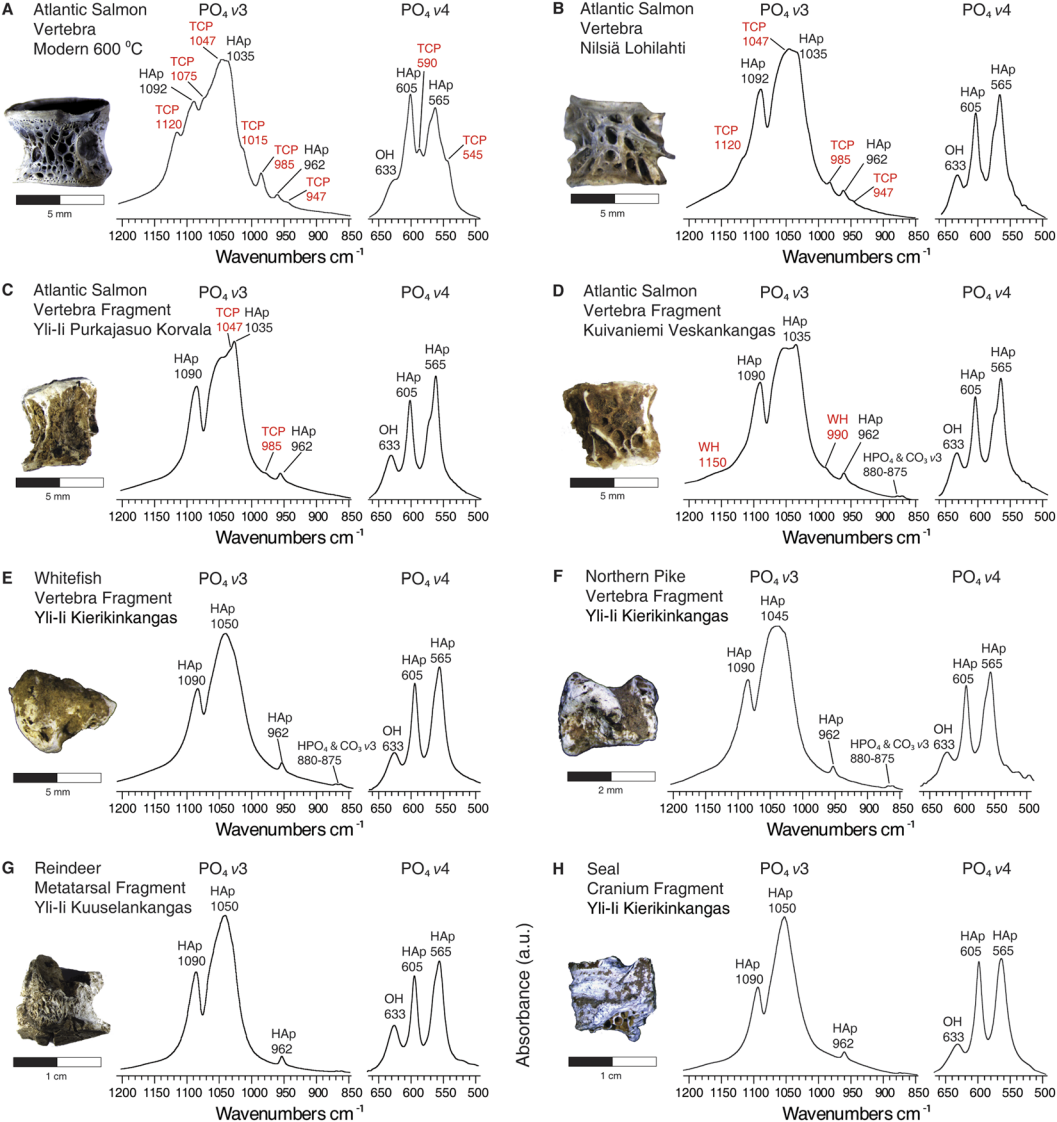
Whitlockite and beta magnesium tricalcium phosphate minerals documented exclusively in the infrared spectra of archaeological Atlantic salmonid bone. *v*3 and *v*4 phosphate infrared absorbance bands for modern Atlantic salmonid (**A**) Atlantic salmonid vertebrae from the archaeological sites of Nilsiä Lohilahti (**B**), Yli-Ii Purkajasuo Korvala (**C**), and Kuivaniemi Veskankangas (**D**), followed by comparative specimens of archaeological whitefish (**E**), northern pike, (**F**), reindeer (**G**), and seal (**H**). Wavenumbers (cm^−1^) for each absorbance band are specified. Bands for whitlockite and beta magnesium tricalcium phosphate are labelled in red. Abbreviations: PO_4_ = phosphate; HAp = hydroxylapatite; HPO_4_ = hydrogen phosphate; CO_3_ = carbonate; OH = hydroxyl; WH = whitlockite; TCP = beta magnesium tricalcium phosphate.

We examined three of the six Atlantic salmonid (*Salmo salar*) bones recovered from the whole of Finnish prehistoric archaeology^8^. The bones, all of which were burned, came from the Nilsiä Lohilahti, Yli-Ii Purkajasuo Korvala, and Kuivaniemi Veskankangas sites (Fig. 1B and Supplementary Information 1C). Bone mineralogy was determined using FTIR. We identified the closely related calcium-magnesium phosphate minerals whitlockite (WH: Ca_9_Mg(HPO_4_)(PO_4_)_6_) and beta magnesium tricalcium phosphate (βMgTCP: Ca_8_Mg (PO_4_)_6_) (compare Fig. 2A with Fig. 2B–D*;* Supplementary Information 2A). The formation of these minerals primarily owes to the Ca^2+^ deficient, Mg^2+^ rich nature of the hydroxylapatite (HAp: Ca_10_(PO_4_)_6_(OH)_2_) mineral in these bones^16–30^. This finding demonstrated that new minerals formed in burned Atlantic salmonid bones can preserve in acidic northern archaeological contexts. We also identified HAp and carbonate (CO_3_). Based on the characteristics of these components, we estimated that the bones were burned at temperatures between 600 °C and 800 °C (Table S1 and Supplementary Information 2B).

To further assess the viability of these mineralogical proxies, archaeological specimens of whitefish (*Coregonus lavaretus*), northern pike (*Esox lucius*), bream, (*Abramis brama*), wild reindeer (*Rangifer tarandus*), and harp seal (*Phoca groenlandica*) were selected for comparative analyses. These species were chosen because they were important resources among the prehistoric populations of northern Finland^8,12^. Whitefish, in particular, appears to have been a very important fish resource. Three quarters of the salmonid bone finds from the entirety of Finnish prehistory are whitefish^8^. Whitefish, northern pike, a cyprinid fish, and harp seal were also represented in the archaeofaunal assemblage we recovered from the Yli-Ii Kierikinkangas case study site *via* fine sieving. Neither WH nor βMgTCP were present in any of these samples (Figs 2E–H and S1). These results suggested that WH and βMgTCP are valuable proxies for burned Atlantic salmonid bone, and they did not form post-depositionally, as they were absent in the other archaeological faunal remains.

**Figure 3:**
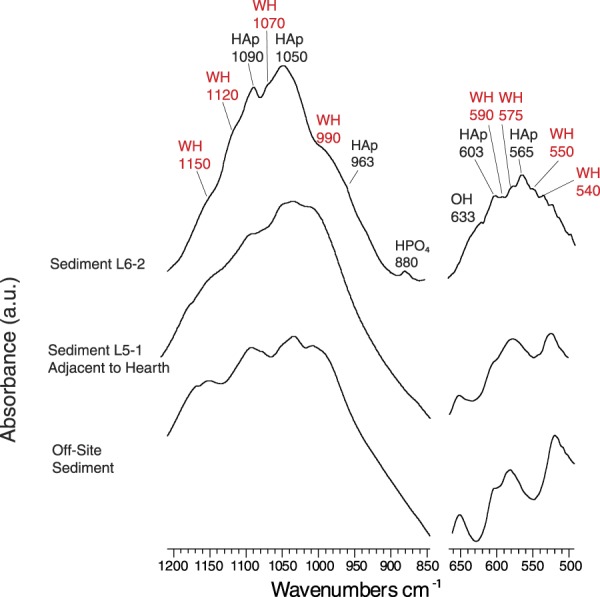
Whitlockite discovered in hearth sediments from the Yli-Ii Kierikinkangas archaeological site (Finland). Examples of infrared spectra for a whitlockite containing bone fragment, archaeological sediment from outside the hearth context, and control sediment collected outside of the site. Wavenumbers (cm^−1^) for each key absorbance band are specified, with whitlockite bands labelled in red. Abbreviations: HAp = hydroxylapatite; HPO_4_ = hydrogen phosphate; OH = hydroxyl; WH = whitlockite.

Next, we tested for the presence of WH/βMgTCP in an archaeological hearth where salmonid remains may have been discarded in prehistory. Sediments collected from a domestic hearth feature inside a pit-house at the 5,600 year old Yli-Ii Kierikinkangas site in northwestern Finland were examined under a stereomicroscope to identify bone fragments (Fig. 1B). Dozens of black, brown, white, and grey particles of what appeared to be bone were selected for mineralogical analysis by FTIR. Several black bone particles (0.5–2 mm in diameter) were found to contain both WH and crystalline HAp (Figs 3, S2, S3D and Supplementary Information 2C)^16–19,30^. HAp crystallinity could not be assessed using the infrared splitting factor (IRSF), as the presence of WH bands distorted the phosphate (PO_4_) *v*4 band required to make the calculation (see Materials and Methods and Supplementary Information 2B). However, both a leftward shift and split in the PO_4_ *v*3 infrared absorbance band were suggestive of somewhat crystalline HAp^16,24^. This confirmed that the examined particles are bone. We contend that the WH discovered in this context originated from burned Atlantic salmonid bone, or possibly burned brown trout bone, considering the physiological similarities of these salmonids. The mineral was exclusively found in bone particles from the archaeological hearth and not in nearby archaeological sediments, off-site sediments, or any of the archaeological bones from the other tested species (Figs 2E–H, S1 and S4).

**Figure 4:**
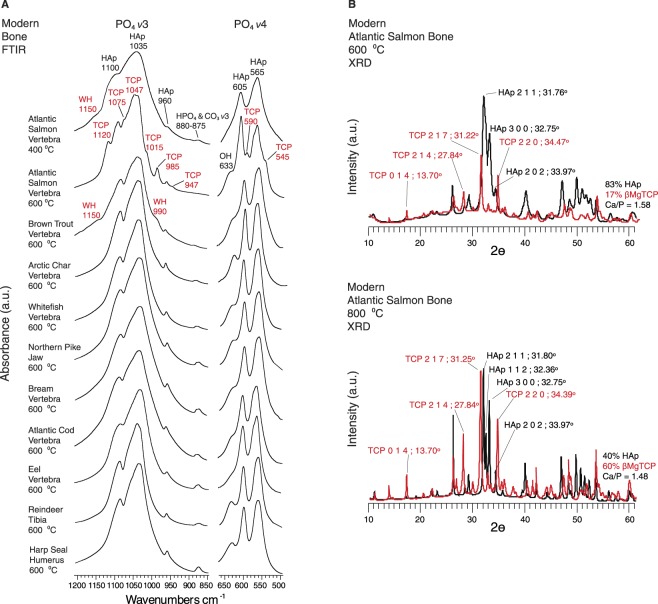
Mineralogical phases in the infrared spectra of burned modern bones. (**A**) Infrared spectra from Atlantic salmonid, brown trout, Arctic char, whitefish, pike, bream, Atlantic cod, eel, reindeer, and seal bone burned at 600 °C. The *v*3 and *v*4 phosphate mineral absorbance bands are displayed. Wavenumbers (cm^−1^) are specified, and bands for the neoformed minerals are labelled in red. Note the marked mineralogical difference between heated Atlantic salmonid/brown trout and the other bones. (**B**) Rietveld refined diffractograms for Atlantic salmonid bone burned at 600 °C and 800 °C showing the percentage of each mineral phase present and the calcium to phosphate ratio (Ca/P). Key reflectance peaks are labelled with their Miller Indices and 2⊖ diffraction angles. Note that the higher the burning temperature the more beta magnesium tricalcium phosphate forms. Abbreviations: PO_4_ = phosphate; HAp = hydroxylapatite; HPO_4_ = hydrogen phosphate; CO_3_ = carbonate; OH = hydroxyl; WH = whitlockite; TCP = beta magnesium tricalcium phosphate.

We conducted burning experiments with modern bones from Atlantic salmonid, brown trout/sea trout (*Salmo trutta/Salmo trutta L*.), whitefish, Arctic char (*Salvelinus alpinus*), northern pike, bream, Atlantic cod (*Gadus morhua*), eel (*Anguilla rostrata*), harp seal, and reindeer to clarify these findings. WH and βMgTCP were identified in Atlantic salmonid bone burned at temperatures as low as 400 °C and 600 °C respectively (Fig. 4A and Supplementary Information 2A). Large amounts of βMgTCP were formed between 600 °C and 800 °C (Fig. 4B). Brown trout produced WH at 600 °C. It also produced large amounts of βMgTCP at 800 °C. No mineral phase changes were observed in any of the other bones burned at 600 °C (Fig. 4A). Seal and reindeer bones did not form WH or βMgTCP at any temperature, yet negligible amounts of βMgTCP were detected in whitefish, Arctic char, northern pike, bream, and Atlantic cod burned at 800 °C. HAp in sequentially heated control bones became more crystalline. Throughout the heating process, increasing IRSF values obtained from the PO_4_ *v*4 band of the bones not forming new minerals indicated progressive growth in HAp crystal sizes and improved atomic order^24^.

Our results also showed that modern Atlantic salmonid bones burned at 600 °C and above had high βMgTCP concentrations and low hydroxyl (OH) concentrations. Conversely, the archaeological specimens were low in βMgTCP and high in OH, suggesting they experienced βMgTCP losses coupled with significant hydration and hydroxylation after burning (compare Fig. 2A with Fig. 2B–D)^25–29^. IRSF values between 5.6 and 6.8 also indicated that their HAp components were highly crystalline. These results suggested that post-depositional hydration caused βMgTCP to transform into crystalline HAp. This is suspected to be a key mechanism involved in the preservation of burned Atlantic salmonid bone fragments/particles and associated neoformed minerals.

We conducted a set of hydration experiments to examine the effects of neutral (distilled H_2_O), alkaline (NaClO), and acidic (HCl) solutions on modern burned fish bones. Mineral characterization with XRD, FTIR, and µCT allowed us to identify three key factors affecting the preservation of burned Atlantic salmonid bone and associated neoformed minerals: (1) the large amount of βMgTCP produced when burned, (2) the reformation of HAp upon hydration, and (3) increases in HAp density and crystallinity.

**Figure 5:**
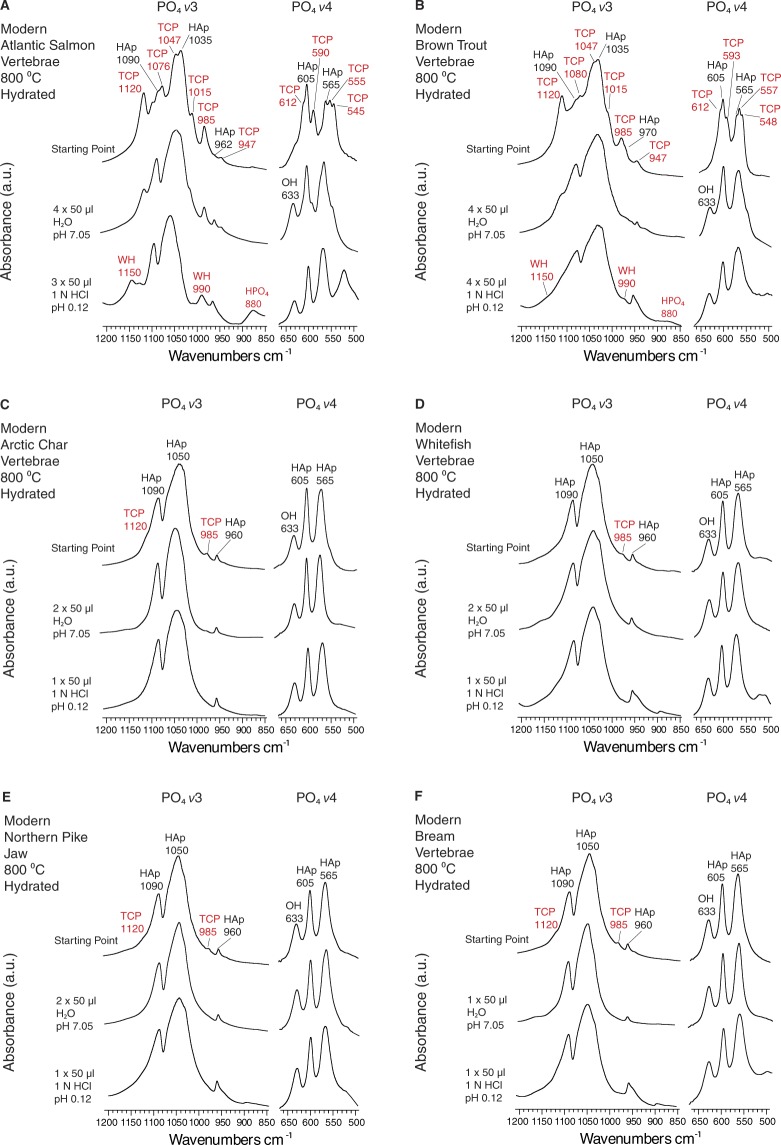
Infrared spectra showing alterations of the phosphate component in burned and hydrated fish bone. *v*3 and *v*4 phosphate absorbance bands for modern Atlantic salmonid vertebrae (**A**), brown trout (**B**), Arctic char (**C**), whitefish (**D**), northern pike (**E**), and bream (**F**) burned at 800 °C. The uppermost spectra in each panel are the experiment starting points of burned but not hydrated bone, while the lower spectra are the results of 50 µl treatments with the designated solution. Wavenumbers (cm^−1^) for each absorbance band are specified, and bands for whitlockite and beta magnesium tricalcium phosphate are labelled in red. Note that beta magnesium tricalcium phosphate bands decrease and hydroxylapatite bands increase upon hydration, and that WH reformed in Atlantic salmonid and brown trout following hydration with acidic solution. Additional details are presented in Supplementary Information B. Abbreviations: PO_4_ = phosphate; HAp = hydroxylapatite; OH = hydroxyl; WH = whitlockite; TCP = beta magnesium tricalcium phosphate; H_2_O = water; HCl = hydrochloric acid.

**Figure 6:**
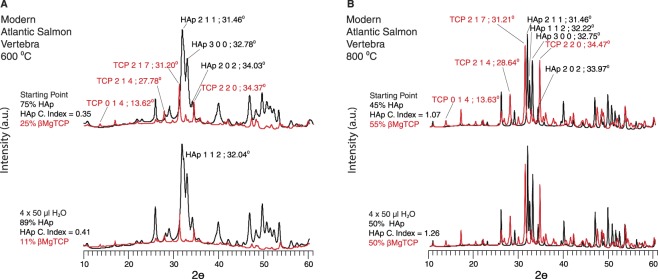
Powder x-ray diffractograms demonstrating the formation of crystalline hydroxylapatite in experimentally burned and hydrated Atlantic salmonid vertebrae. Rietveld refined diffractograms for modern salmonid vertebrae incinerated at 600 °C (**A**) and 800 °C (**B**) then hydrated with distilled water are displayed. Hydroxylapatite and beta magnesium tricalcium phosphate phases are represented in black and red respectively. The upper diffractograms show the starting points of the experiments, while the lower ones are the experiment results after four 50 µl hydration treatments. Percentages of each mineral phase and crystallinity indices for hydroxylapatite are listed. Key reflectance peaks are labelled with their Miller Indices and 2⊖ diffraction angles. Note that HAp crystallinity is considerably higher in hydrated bone initially burned at 800 °C. Abbreviations: HAp = hydroxylapatite; TCP = beta magnesium tricalcium phosphate; H_2_O = water.

Rietveld refined diffractograms showed that burned Atlantic salmonid bone contained large amounts of βMgTCP. Higher temperature burning produced larger amounts, with samples burned at 800 °C containing as much as 60% βMgTCP (Fig. 4B). Brown trout contained similar amounts, given the similarities shared across the infrared spectra for both these specimens and Atlantic salmonid. Hydration with neutral and acidic solutions both reduced βMgTCP concentrations in modern burned Atlantic salmonid and brown trout bones (Figs 5A,B, 6 and Supplementary Information 2D). The degree of loss was connected to the concentration initially produced. Atlantic salmonid bones burned at 600 °C and 800 °C had βMgTCP concentrations of 25% and 55% respectively. Hydration with four 50 µl surface treatments of H_2_O caused drastically different βMgTCP decreases in these samples. We recorded a 56% reduction in the former and a much more modest 9% decrease in the latter. These findings demonstrated that the higher βMgTCP concentration, the more will remain following hydration (Fig. 6 and Supplementary Information 2D). As stated above, small amounts of βMgTCP were formed in modern whitefish, Arctic char, northern pike, bream, and Atlantic cod samples burned at 800 °C. Unlike the modern Atlantic salmonid and brown trout bones, the samples from these other fish completely lost their βMgTCP components after two treatments with H_2_O and one or two treatments with HCl (Figs 5C–F and S5).

Rietveld refined diffractograms and the HAp crystallinity index (CI) showed that βMgTCP reductions in Atlantic salmonid bone were accompanied by increases in both HAp concentrations and crystallinity (Fig. 6). HAp crystallinity could not be calculated for modern burned samples using infrared spectra because the presence of large quantities of βMgTCP altered the characteristics of the HAp PO_4_ *v*4 absorbance band. We therefore used Rietveld refined diffractograms to separate the reflectance peaks for each mineral and thus assess HAp CI without compromise. After hydration with four 50 µl surface treatments of distilled H_2_O, the sample burned at 600 °C formed the HAp 1 1 2 reflectance peak at 32.04° 2⊖. Several additional peaks such as the 2 0 2 at 34.03° 2⊖ became more pronounced and sharper. These changes qualitatively suggested that the mineral became more crystalline with the loss of βMgTCP. The XRD CI increased from 0.35 in the untreated burned bone to 0.41 in the hydrated sample, confirming a 17% increase in HAp crystallinity accompanying the large 56% decrease in βMgTCP (Fig. 6A). Comparatively, bones burned at 800 °C then treated with the same hydration procedure showed smaller decreases in βMgTCP concentrations yet similar increases in HAp CI values. CI values for this sample before and after hydration were 1.07 and 1.26 respectively. This 18% increase in HAp crystallinity occurred alongside a comparatively small 9% decrease in βMgTCP (Fig. 6B).

The formation of large, well-ordered HAp crystals is expected to increase bone mineral density. Our µCT results showed that the tested modern air-dried Atlantic salmonid vertebra had a tissue mineral density (TMD) of 439 mg HAp/cm^3^, which is consistent with µCT TMD results reported for zebrafish and with volumetric mass densities reported for chinook salmonid (Fig. S6)^13,31^. Small increases in mineralization were documented in burned (TMD = 482 mg HAp/cm^3^) and burned-and-hydrated samples (TMD = 501 mg HAp/cm^3^). Bone density studies report that changes as small as 5–8% can increase bone strength by over 60%^32^. The change in TMD between the burned and burned-and-hydrated samples was 3-4%, which shows the trend expected for the early stages of hydration in archaeological contexts. The tested archaeological Atlantic salmonid vertebra had a significantly high TMD of 930 mg HAp/cm^3^, showing the much more pronounced effect of prolonged hydration on mineral density in the archaeological burial environment.

Based on these archaeological and experimental findings, we propose that post-depositional hydration promotes the transformation of a fraction of the βMgTCP into dense crystalline HAp. This more chemically stable HAp agglutinates around remaining βMgTCP grains, in turn reducing both mineral surface area and overall bone porosity^25–29^. These characteristics will slow mineral dissolution and/or complete βMgTCP transformation into HAp by limiting the rate and degree of solution movement throughout the bone structure. This is strongly supported by our observation that modern Atlantic salmonid bones burned at 800 °C and hydrated with H_2_O had modest reductions in βMgTCP coupled with increases in HAp crystallinity (Fig. 6B). The lack of organic matter in highly burned bone will also reduce microbial activity, further improving burned bone survivorship. For these reasons, burned salmonid bone fragments and associated neoformed minerals are expected to have better preservation potential than highly organic, unburned bone.

Additionally, modern Atlantic salmonid and brown trout bones burned at 600 °C to 800 °C and then hydrated with acid solution became darker in colour, and they presented infrared absorbance bands at 1,150 cm^−1^ and 880 cm^−1^, specifically the samples burned at 800 °C (Figs 5A,B and S3A,B).This suggested that WH and hydrogen phosphate (HPO_4_) may also reform in highly burned Atlantic salmonid and brown trout bones that have been exposed to acidic soil/sediment solution. It appears that the introduction of acidic solution can cause some of the βMgTCP to hydrolyze into OH, HPO_4_, and Ca^2+^ deficient HAp^25^. This would cause the remaining calcium-magnesium phosphate mineral to take on the form of the WH polymorph because of the reintroduction of HPO_4_, which explains the presence of OH, HPO_4_, and WH in one of the studied archaeological Atlantic salmonid bones, as well as the presence of black, WH containing bone particles in the Yli-Ii Kierikinkangas hearth (Figs 2D and 3).

We expect large amounts of βMgTCP produced in individual Atlantic salmonid and brown trout bones, or present in deposits containing dense concentrations of burned Atlantic salmonid/brown trout bone remains, to be well-preserved under neutral conditions and preserved to a small degree under acidic conditions. WH may reform out of βMgTCP in acidic soils/sediments. Small amounts of βMgTCP formed in bones from other species are not expected to preserve in archaeological contexts.

## Discussion and Conclusion

Our findings have exciting implications for northern hunter-gatherer archaeology and, more broadly, for studies of dynamics in human-riverine-coastal ecological networks. First and foremost, the identified mineral proxies advance research on ancient northern salmonid fisheries from heuristics into hard evidence, improving our ability to answer questions concerning where and when salmonids have been utilized in northern prehistory. The people living at the Yli-Ii Kierikinkangas site are suspected to have harvested this resource; yet, before our study, no classifiable bone specimens confirming this have ever been recovered^8^. Calcium-magnesium phosphates were not preserved in any of the morphologically classifiable animal bones recovered from the site. The only other documented source for these minerals in Finnish archaeological contexts comes from the three rare Atlantic salmonid bones analysed for this study. Based on our archaeological and experimental findings concerning the mineralogy of burned fish, seal, and reindeer bones, Atlantic salmonid and/or brown trout are the most reasonable sources for the WH identified in the hearth context we studied. Our discovery of morphologically classifiable whitefish bone fragments and bone particles containing WH and crystalline HAp in this hearth provides the first unequivocal evidence for the disposal of salmonid remains at this important site.

Evidence for both sealing and salmonid fishing at Yli-Ii Kierikinkangas offers insight into seasonal resource use and residential mobility. The prevalence of harp seal bone at the site suggests a reliance on this resource. Optimal sealing seasons in the northern Baltic Sea are greatly dependent on ice conditions. In open waters, sealing is much more difficult (and dangerous), and the catch would not be as reliable as during the breeding season in early spring. Harp seal is the most commonly identified seal species in (Sub-)Neolithic archaeofaunal assemblages in northern Ostrobothnia^33^. The life and breeding habits of this species are highly dependent on ice conditions. Ethnographic evidence suggests that the peak period for harvesting on the ice was during the late winter/early spring breeding season, when seal behaviour was highly predictable. Archaeological research at more southerly sites in Finland, where bone preservation is much better, also suggests that yearlings and adults were hunted at breeding grounds during late winter/early spring^34^.

The availability of Atlantic salmonid and anadromous brown trout vary seasonally. During the historic period, Atlantic salmonid availability in the Iijoki River typically peaked during spawning runs from June through November^8^. The anadromous brown trout (sea trout) found in northern Fennoscandia rivers are known to have migration behaviours similar to Atlantic salmonid^35^. They also move very little during the winter months, typically staying in deeper pool habitats^36^. Anadromous whitefish were likely available during the late winter and throughout the summer and fall^8^. Considering both anadromous fish behaviour and the difficulties of winter river fishing, we propose that salmonid fishing at Yli-Ii Kierikinkangas was a warmer season activity, likely spanning spring through fall.

Based on the seasonal availability of these animals, as well as the time/labour investment and logistics involved in preparing both sealing and fishing gear, harvesting, processing, and readying potential surpluses for storage, evidence for both sealing and salmonid fishing at Yli-Ii Kierikinkangas suggests that people spent most of, if not the entire year living at this village^37^. This offers additional support to previous reports of multi-season occupations at sites along the north shore of the Iijoki River, and at Yli-Ii Kierikinkangas in specific. Pit-house architecture, the presence of smaller pits likely used for storage, a high diversity in tool types, and trade goods have been cited as evidence for longer term occupations at these sites^8,38^.

Longer term or permanent occupations at Iijoki River villages have been explained as an adaptive response to increasing population densities, isostatic uplift, and a contracting coastline throughout the (Sub-) Neolithic^38^. It is possible that these coinciding shifts placed an increasingly high premium on economically advantageous locations along both the northwest coast and the Iijoki River. Yli-Ii Kierikinkangas is one such location. The camp was located in an estuary habitat, providing unfettered access to harp seal winter breeding grounds. The narrows formed by a large river island directly adjacent to the village would have also been a profitable location to position wooden weir structures and/or guiding fences for salmonid harvesting^8,9^. For these reasons, this location would have been highly valuable, likely circumscribed and protected, with ownership being passed through family lineages. Such a claim to this land would have demanded more permanent occupations^38^.

New investigations of the palaeo-morphology of this part of the river, specifically aiming to determine whether the area was suitable for positioning wooden fishing structures, would provide additional support for this scenario. More importantly, the remains of the wooden weir and lath screen structures themselves would provide excellent evidence for salmonid harvesting at Yli-Ii Kierikinkangas. Only a few km west of the site, excavations of the wetlands flanking the Purkajasuo site revealed well-preserved screen structures made from pine laths bound with birch bark strips^8–10^. This site also yielded one of the Atlantic salmonid vertebrae examined in this study. Based on our discoveries of salmonid remains at Yli-Ii Kierikinkangas, we propose that the wetlands immediately south of the camp should be investigated for the presence of wooden fishing structures. The identification of calcium-magnesium phosphate proxies for burned salmonid bone in hearths at other sites would be useful for targeting additional river-side locations that should be searched for wooden fishing structures. The identification of these fishing technologies near Yli-Ii Kierikinkangas, and other sites and wetlands, will contribute to broadening our understanding of the interplay between sedentism, salmonid fishing, and changing social and natural landscapes along the Iijoki River.

Atlantic salmonid and brown trout share many behavioural, physiological, and morphological similarities^39^. The results of our experiments suggest that their bone composition is also very similar, as they both undergo the same unique mineral phase transformations when burned at temperatures between 600 and 800 °C. Our future work on this topic will aim to clarify the mechanisms dictating the degree of βMgTCP formation in heat treated bone from different salmonid species. It is possible that the anadromous behaviour and physiology of these fishes play important roles in this regard. Differences in bone fat, Ca^2+^, Mg^2+^, collagen, and cartilage contents may be at the centre of this. It is known, for instance, that Atlantic salmonid lose a fraction of their Ca^2+^ stores during spawning because they stop feeding during this freshwater stage of their life cycle^20,40^. As a result, the skeleton looses Ca^2+^ and takes on a more cartilaginous form^20^. It is also widely agreed that Mg^2+^ is a key factor in the formation of βMgTCP in bone and HAp bioceramics^17–30^. Both fishes also experience fat and protein losses during winter and rapid increases during spring^39^. The influences of these characteristics on mineral transformation *via* burning in both Atlantic salmonid and brown trout bone require additional investigation. The formation of calcium-magnesium phosphates in pacific salmonids should also be investigated. The presence of these minerals in Alaskan and High Arctic contexts may be useful for identifying the use of salmonid resources among pioneering people in these regions.

We conclude that calcium-magnesium phosphate proxies will expand our knowledge of salmonid harvesting activities at different times and places. Mineralogical profiling of soils and sediments, however, is uncommon in northern archaeology. We propose that FTIR spectroscopy/micro-spectroscopy will provide inexpensive, high throughput ways of mapping sediment mineralogy at northern archaeological sites. These novel mineral proxies are expected to contribute to clarifying regional and temporal dynamics in northern human-salmonid ecologies.

## Materials and Methods

### Laboratory Experiments

We burned bones from modern Atlantic salmonid (*Salmo salar* n = 3), bones from other salmonids, specifically brown trout (*Salmo trutta* n = 3; anadromous sea trout variety *Salmo trutta L. n = 3*), whitefish (*Coregonus lavaretus* n = 2) and Arctic char (n = 3), along with bream (*Abramis brama* n = 2), northern pike (*Esox lucius* n = 2), Atlantic cod (*Gadus morhua* n = 3), eel (*Anguilla rostrata* n = 1), harp seal **(***Phoca groenlandica* n = 2), and reindeer (*Rangifer tarandus* n = 3) to determine the effects of heating on their mineralogical characteristics. Fish vertebrae, reindeer tibia, and seal humeri were used. Bones from different animals of each species were used to ensure reproducibility. Defleshing and cleaning of modern bones were done under warm running water using a scalpel and nylon brush. Samples were air-dried, and any remaining non-bone tissue was removed using a scalpel. Burning was done in an electric laboratory furnace (Thermolyne F6000, Thermo Scientific) in open atmosphere through a complete sequence of 100, 200, 300, 400, 500, 600, 700, 800, 900, and 1,000 °C for 1 h at each temperature. The oven was preheated to the desired temperature before samples were introduced. Specimens were removed from the oven after each interval, subsampled, and placed back into the preheated oven for treatment at the next temperature. Sample surfaces were scraped with a scalpel to produce powders for Fourier transform infrared spectroscopy analyses (FTIR). Whole samples were used for x-ray diffraction (XRD) analyses.

We examined the effect of hydration on modern burned fish bone to determine whether this process is related to the preservation or diagenesis of burned bone and associated neoformed minerals. Duplicate hydration experiments were undertaken using neutral, alkaline, and acid solutions: distilled water (H_2_O, pH = 7.05), sodium hypochlorite (NaClO, 5%, pH = 11.87), and hydrochloric acid (HCl, 1 N, pH = 0.12) respectively. We used these solutions not to simulate archaeological conditions, but to determine whether the neoformed minerals could be easily lost, and to ensure that the results could be easily replicated in any other laboratory setting.

Specimens burned at 600 °C, 800 °C, and 1,000 °C were used to understand changes in mineral characteristics caused by hydration after the initial formation of βMgTCP (600 °C) and when the βMgTCP concentration increases (800 °C and 1,000 °C). Samples were divided into nine glass Petri dishes, one dish for each temperature and solution (e.g. 600 °C H_2_O, 600 °C NaClO, HCl 600 °C, etc.). Each experiment was completed over a four day period. On the first day, samples were burned, immediately characterized by FTIR, then immediately hydrated with 50 µl of solution delivered to the sample surface *via* automatic pipette. Samples were left to absorb the solution and air-dry for 24 h, after which powders were scraped from their surfaces for characterization by FTIR, and then hydrated again. Each sample was treated in the same manner over the next three days.

### Sediment Sampling

Sediments were collected from the Yli-Ii Kierikinkangas archaeological site in northwest Finland (June 2017). Collection took place during the excavation of a pit-house, and specifically focused on the centre of the dwelling where the hearth feature was expected to be located. A 4 m^2^ area was sampled vertically and horizontally. Point samples were taken systematically on a horizontal square lattice grid at 50 cm intervals, as well as opportunistically in areas of interest, such as pockets of charcoal or bone. Sampling was done in this fashion at roughly 5 cm vertical intervals as the excavation progressed. Additional opportunistic sampling was performed adjacent to the initial 4 m^2^ area once the pit-house excavation was expanded. Roughly 150 g of sediment was collected from each location with a clean stainless steel scoop and stored in plastic sampling bags. Seventy-five samples were collected in this manner. Eleven bulk sediment samples totalling 33 l were also collected opportunistically. Six control sediment samples were collected from locations several hundred meters north of the site.

In the laboratory, point samples were air-dried and passed through a geological sieve column separating the sediments into the following sizes: >2 mm, 2-1 mm, and 1–0.5 mm. Fragments having the appearance of charcoal, charred matter, charred bone, or calcined bone in the 2-1 mm size fraction were identified by eye, while particles from the 1-0.5 mm size fractions were identified using a Leica M80 stereo microscope at 2–6x magnification. Overall, 136 specimens were selected for analysis. The bulk sediments were air-dried and sieved using 1 mm and 4 mm mesh sizes to identify bone fragments.

pH measurements were made using 5 g of sediment and 50 ml of calcium chloride solution. The sediment/solution mix was stirred on a shaker table for 30 min then left to settle for an additional 30 min. Measurements were taken from the supernatant using a Jenway 3540 pH meter. The meter was calibrated with pH buffer solutions at pH 4, 7, and 10. Measurements were taken at 22.0 °C.

### Mineralogical Characterization

All of the bone and sediment samples were analysed using a Nicolet iS5 (Thermo Scientific) FTIR spectrometer to characterize their mineral components. Archaeological fish, reindeer, and seal bones were studied for comparison and experimental control. These included: whitefish, northern pike, cyprinid, and two teleosts from the site of Yli-Ii Kierikinkangas (n = 10), 35 seal bone samples (15 from Yli-Ii Kierikinkangas, 10 from Yli-Ii Purkajasuo Korvala, and 10 from Kuivaniemi Veskankangas), and nine reindeer samples (4 from Simo Tainiaro and 5 from Yli-Ii Kuuselankangas) (Supplementary Information 1C). Loose sediments were gently brushed from the archaeological bone samples before testing. For all experimental and archaeological samples, a roughly 1:20 ratio of sample to potassium bromide (KBr) was homogenized and ground using an agate mortar and pestle. The blend was evenly dispersed inside a steel die and pressed at 2,000 kg for 60 s. Spectra were averaged from 32 scans in the 4,000 to 400 cm^−1^ region at 4 cm^−1^ resolution. Mineral components were identified through comparison with published data^16–19,21–30^.

Modern Atlantic salmonid bones were analysed by XRD to confirm and quantify mineral phases. Samples were ground into powders with an agate mortar and pestle. These were evenly dispersed in a glass sample holder with 20 mm × 20 mm × 0.5 mm of square space and compacted with a spatula. Diffractograms were collected using a Rigaku Miniflex 600. The instrument ran at 30 kV and 10 mA and was equipped with a Cu x-ray tube (λ = 1.5418 Å), vertical goniometer, Kβ foil filter, and an SC-70 detector. The incident slit was 1.250° and the length of the limiting slit was 10 mm. Receiving slits 1 and 2 were 1.250° and 0.3 mm respectively. Incident and receiving soller slits were both 2.5°. Data was collected at 4°/min in the angular 2⊖ range of 0 to 60° with a step width of 0.02°.

Mineral phases were quantified through Rietveld refinement using Profex software v. 3.9.2. The procedure fit a model of hypothesized mineral phases to the observed diffractogram using non-linear least squares regression. Model quality was assessed using the chi-squared goodness-of-fit statistic. Values closer to 1 indicated optimal correspondences between observed and refined diffractograms. Weight percentages of Ca, Mg, and P were automatically calculated by Profex using crystallographic indicators of atomic site occupancies and the atomic weights of each site’s species^41^. These were converted into molar masses to calculate element molar ratios, specifically the Ca/P, which is useful for identifying Ca deficiencies (<1.67) and classifying different calcium-phosphate minerals^18^.

Bone HAp crystallinity was assessed using both FTIR and XRD. Concerning FTIR, the HAp phosphate *v*4 absorbance band was used to define crystallinity, specifically by calculating the IRSF, which divides the sum of the 605 cm^−1^ and 565 cm^−1^ band heights by the height of the valley separating them. This value provides an indication of short range atomic order/disorder in the crystal lattice of the HAp mineral^24^. This is the most widely used means of calculating the crystallinity of HAp from FTIR spectra. Working with this approach makes our data comparable to results obtained across several disciplines. Burning increases atomic order and crystal sizes in the HAp, making the mineral more crystalline, and eventually causing grain agglutination, in turn providing larger IRSF values. For example, values for modern unaltered mammal bone typically have IRSF values between 2.5 and 3.0. Values for moderately crystalline bone range between approximately 3.6 and 4.5, while those for highly crystalline bone range between 5 and 7^42^.

HAp crystallinity could not be calculated for modern burned Atlantic salmonid bone samples using the IRSF because the presence of large quantities of βMgTCP altered the characteristics of the HAp PO_4_ *v*4 absorbance band. We therefore used Rietveld refined diffractograms to separate the reflectance peaks for each mineral and thus assess HAp crystallinity without compromise. Changes in crystallite sizes and long range atomic order in crystal lattices were identified using the XRD CI. This was calculated using the heights (H) of the 2 0 2, 3 0 0, 1 1 2, 2 1 1 reflectance peaks: CI = Σ {[H 2 0 2, H 3 0 0, H 1 1 2]/H 2 1 1}^43^. The CI is the most commonly used XRD technique for assessing the crystallinity of HAp; as with the IRSF, we used this method to ensure pan-disciplinary data comparability. The CI increases with increasing crystallite size and atomic order. Low levels of crystallinity provide CI values between 0 and 0.28. Values from 0.29 to 0.89 are moderately crystalline, while those above 0.90 are highly crystalline^43^.

We used µCT to determine the bone tissue mineral density (TMD) of four Atlantic salmonid vertebrae samples: modern air-dried, modern burned at 800 °C, modern burned at 800 °C and hydrated with H_2_O, and an archaeological sample from the Finnish site of Nilsiä Lohilahti. We recorded 1,200 projections over 180° with a Zeiss Xradia MXCT-400 instrument (Zeiss x-ray microscopy, Pleasanton, California USA) using a voltage of 40 kV and a current of 200 μA. Under these conditions, the final voxel size of the images was 10.3 μm. The volume reconstruction was made using a filtered backprojection algorithm (Zeiss-Xradia proprietary software).

### Ethics Statement

Fish and seal used in this study were legally purchased from fish markets in Israel, Canada, and Finland. Reindeer bones were sourced from a legal and regulated reindeer farm in Finland. Permits were not required to possess the animal specimens. Animals were not live upon acquisition. No animals were sacrificed for research purposes, nor were any animals used in the study a threatened or endangered species.

## Electronic supplementary material

Supplementary Information

## Data Availability

The key data generated and/or analysed during this study are included in this published article (and its Supplementary Information files). The complete datasets generated during and/or analysed during the study are available from the corresponding author on reasonable request.
