# Supplementary material for: Early Evidence for Northern Salmonid Fisheries Discovered using Novel Mineral Proxies
Source: Sci Rep. 2019 Jan 16;9:147. doi: 10.1038/s41598-018-36133-5 (PMC6335411; doi:10.1038/s41598-018-36133-5)
Supplement: Supplementary file 1 — Supplementary Information [file 41598_2018_36133_MOESM1_ESM.docx]

**Supplementary Information**

**Early Evidence for Northern Salmon Fisheries**

**Discovered using Novel Mineral Proxies**

D.H. Butler^*^, S. Koivisto, V. Brumfeld, and R. Shahack-Gross^*^

* To whom correspondence should be addressed: D.H.B. email: dhbutler@ucalgary.ca;

R.S.-G. email: rgross@univ.haifa.ac.il

**This Supplementary file includes:**

**Supplementary Information 1: Archaeological Background**

1. **Prehistoric Fishing in Northern North America and Scandinavia**
2. **Salmonids in Finnish Archaeology**
3. **Study Sites in Finland**

**Supplementary Information 2: Extended Results**

1. **Mineralogical Phase Transformation in Atlantic Salmon Bone**
2. **Determination of Heating Temperature in Atlantic Salmon Bone**
3. **Yli-Ii Kierikinkangas Hearth**
4. **Influences of Hydration on Burned Atlantic Salmon Bone Having Different Amounts of βMgTCP**

**Supplementary Figures S1-S6**

**Supplementary Table S1**

**Supplementary References**

**Supplementary Information 1: Archaeological Background**

**A. Prehistoric Fishing in Northern North America and Scandinavia**

Little is known about salmonid fishing amongst the earliest people to explore the high, low and sub Arctic regions of North America. At this stage of North American Arctic archaeology, with over 100 years of research, the perceived rarity of fish bones at ancient campsites is deeply embedded in the common knowledge. The scarcity of these materials is obstructing our ability to understand dynamics in northern economies and human-environment interactions^1-4^. The earliest sites documented in Alaska are close to 16,000 years old, and the people living in this region, referred to as eastern Beringia, during this early period engaged in hunting a broad array of animals. High frequencies of large mammal bones suggest they specialized in big game hunting. Few sites of such time depth have yielded fish bones. Less than 30 salmonid bones were discovered at the Broken Mammoth site (∼12,080 BP) in central Alaska^3^, and a small assemblage of unclassifiable fish bones were recovered from the Mead site (∼11,990 BP) in eastern Alaska^4^. The only report of identifiable Pacific salmonid remains in eastern Beringia comes from the Upward Sun River site on the Tanana River in central Alaska. Some 300 Pacific salmon bones were recovered from a hearth context dating to 11,600 years BP. Ancient DNA extracted from two bones confirmed chum salmon as the species of origin^1^.

Arctic char is the most widely available salmonid in the eastern North American Arctic, yet little can be confidently said about their use among pioneering Independent I and Pre-Dorset groups. The Independence I people were the earliest explorers of the Canadian High Arctic. They entered this region roughly 4,500 years ago and rapidly migrated across the archipelago into Greenland. Many Independence I sites in Canada have low numbers of preserved animal bones. Fish bones are largely absent in these contexts. For instance, fish bones have not been reported from key Independence I sites investigated on Devon Island^5, 6^. The role of Arctic char in the economies of the Independence I people of the High Canadian Arctic remains unresolved. Arctic char bones have been recovered from several Independence I sites in High Arctic Greenland, yet very few bones of any animal species are found in the south, which likely owes to a preservation issue^7^.

Roughly 4,500 years ago, the Pre-Dorset people were the first to travel across the lower latitudes of the eastern Canadian Arctic^5^. Arctic char is also assumed to have been an important resource among these people. Reports on their inclusion in Pre-Dorset economies cite evidence such as ethnographic accounts for the importance of the resource during the historic period, its abundance in lakes/rivers near archaeological sites, the presence of rock structures interpreted as fishing weirs, and very small bone assemblages^8^. The Large Lakes region of southern Baffin Island, for example, is considered an excellent place to fish for char. However, excavations at four sites along the shore of Mingo Lake produced very few fish bones. The largest assemblage from these sites contained over 18,000 animal bones, only nine of which were identified as Arctic char. Only one unclassifiable fish bone was retrieved from the other three site^9^. Similar to this situation, a rich archaeological record has been investigated on Igloolik Island, yet a meagre number of fish bones have been reported. Even sites interpreted as summer camps, the season during which fishing is expected, typically produce little or no evidence for fishing. Well over 100 archaeological features have been recorded at the Lyon Hill site, yet not a single fish bone was reported. At a nearby site, just three fish bones were recovered from the excavations of six dwelling structures^10^. Similarly, at the Crane site on the Cape Bathurst Peninsula in the western Canadian Arctic, a faunal assemblage of close to 40,000 bones yielded just a few fish bones. North of here, only one fish bone was found at the Lagoon site on Southern Banks Island^11^. These finds are considered typical of Pre-Dorset sites^10^.

Reports of Arctic char bone, and fish bone in general, from Dorset (~2,500 to 500 BP) and Thule (~1,100 to 500 BP) sites are equally rare^12-14^. Foundational investigations of several Dorset sites on Victoria and Banks Islands in the central Arctic do not report on the presence of fish bones^11^. To the east, the Foxe Basin has been extensively researched, and is considered the core area of the Dorset world. Investigations on Igloolik Island have made significant contributions to understandings of economic dynamics in this region. Numerous Dorset sites have been excavated, yet few contain fish bones. Where char or other fish bones were documented, they were typically found in such low numbers that they were grouped with other sparsely found animal bones, such as birds and polar bear, to create a faunal category labelled ‘other’^10^. Returning to Mingo Lake in the eastern extent of the Dorset core, the investigated Dorset component contained hundreds of animal bones, just nine of which were identified as fish^15^. The Tayara site, in the southern extent of the core, is one of the key sites that contributed to the definition of the Early Dorset period. No fish bones were reported^16^. Extensive research has also been undertaken on the role of fish resources among the Thule people. Tools interpreted as fishing gear are somewhat common, yet the fish bones that would provide direct evidence for fishing are largely absent from most Thule sites^12^.

Among the ancient First Nations cultures of the northeastern Canadian Shield, specifically the Northern Plano (~8,000 - 7,000 years BP), Shield Archaic, (~6,500 - 3,500 years BP), and Taltheilei (~2,600 - 200 years BP) cultures, fish is commonly described as a secondary resource, one typically sought during times of caribou scarcity^17^. As a result, little attention has been given to the use of fish by these people. Archaeological remains are also scant. A comprehensive synthesis of over 1,000 sites in the Barrenlands region of the central subarctic mentions few fish bone discoveries^17^. For example, several Taltheilei sites investigated off the west coast of Hudson Bay, despite their positioning on the shores of a productive lake, yielded no fish bones^18-20^.

Across the Arctic Ocean, in Scandinavia, the salinity and ecological characteristics of the Baltic Sea had major influences on the distribution of various fish species. After the last deglaciation, the first fish species entering the Baltic Basin were Atlantic salmon (*Salmo salar*), trout (*Salmo trutta*), arctic char (*Salvelinus alpinus*), and whitefish (*Coregonus lavaretus*)^21^. Salmonidae, pike (*Esox lucius*), and perch (*Perca fluviatilis*) were also present in the Baltic Ice Lake, predecessor of the Baltic Sea ca. 12,600-10,300 BP^22, 23^. After the ice sheet retreated northwards, various fish species such as bream (*Abramis brama*) and pikeperch (*Sander lucioperca*) moved into the expanding Ancylus Lake and its extensions at around 9,500-8,000 BP^23^. During the Holocene Thermal Maximum, ca. 6,000-2,500 cal BCE, temperatures were a few degrees higher than today, which may have caused some fish species to adapt to warmer biotopes and led to a much wider distribution than today^24-26^. Major rivers and estuaries constituted excellent habitats for the fry of anadromous fish species, mostly Atlantic salmon. In addition, whiteﬁsh and river lamprey (*Lampetra ﬂuviatilis*) migrated to spawn in the rivers draining into the Gulf of Bothnia. Other migratory species were also present, such as grayling (*Thymallus thymallus*) and East European bream (*Vimba vimba*).

The utilization of marine and freshwater fish by Scandinavian populations in prehistory has varied greatly both temporally and regionally. Populations were able to respond to changes in resource availability to target abundant species. Mesolithic subsistence systems in the northern Baltic Sea area have typically been characterized as broad spectrum, focusing on seasonally aggregated resources, especially seal and anadromous fish (even though there is a limited amount of archaeofaunal evidence confirming this), and waterfowl, fur-bearing animals, and reindeer^27^. As in many northern areas around the world, the significance of fish in human subsistence and diet is difficult to investigate due to low quantities of fish bones at archaeological sites. Strong biases caused mainly by poor preservation of archaeofaunal remains and the representation of archaeological materials obscure fishery studies and dietary investigations.

In Western Norway ca. 9,500 cal BCE, the first settlers relied mostly on marine mammals. By 8,000 cal BCE, fishing became more important, and it remained significant until the Late Neolithic, ca. 2,500 cal BCE^28, 29^. A small number of salmonid bones have been found at a few Mesolithic and Neolithic sites in southern and western Norway. At Mesolithic rock shelter sites in western Norway, the preservation conditions for bones are better than at open-air sites. Fish remains at these sites come primarily from the cod family (gadids)^29^. Salmonid remains are scarce, even at sites situated near advantageous salmon fishing locations. They comprise roughly 2 % of the total fish bone taxa. The small number of salmonid bones may be caused by poor preservation conditions, but it may also reflect Mesolithic fishing methods. The rock shelters were utilized for fjord fishing and people primarily fished near-shore using bone hooks^29^.

Fishery studies concerning the early Holocene in Southern Scandinavia (Denmark and southeast Sweden) are greatly affected by eustatic sea level changes and the submergence of extensive coastal areas^30, 31^. Based on a number of preserved sites from the Early and Middle Mesolithic, ca. 10,500-7,500 BP, forager groups utilized aquatic resources at a large-scale. Mass-harvesting and preservation methods for fish were widely applied^32, 33^. The Ertebølle populations of eastern Denmark, ca. 5,400-4,000 BCE, seem to have had a narrower diet breadth focusing mostly on hook-and-line fishing of gadids than those in the west, who also practiced weir fishing of eel (*Anguilla anguilla*) during warm seasons^34^. Eels are present in virtually all fish bone assemblages, with freshwater species being a minor component in the Ertebølle fisheries. A recent study from southern Scania reveals that subsistence strategies likely became more reliant on fish during the Mesolithic^33^. Salmon bones are not common in southern Scandinavian Mesolithic sites, even in areas with good organic preservation. Based on current knowledge, salmon do not appear to have been of significance among people in the southern and western parts of the Baltic, whereas its importance in regions further north, in northern Sweden, Norway, and Finland, has not been adequately studied^31-33^.

Considering the entire breadth of archaeological research in the northern regions of North American and Scandinavia, fish bone is one of, if not the most, poorly represented types of faunal evidence, particularly at sites dating to earlier time periods. As a result, fishing is one of the most misunderstood aspects of past economics and human-environment relationships in these regions. Larger assemblages of fish bones have been recovered from more recent sites in northern North America, yet these exceptional sites are few, and they only inform on the later developmental stages of human-fish resource interactions^35-39^. The developments leading up to the use of fish at the scale represented at these richer, more recent sites have not been well documented because of the lack of direct evidence for fishing through time. Based on available reports, it is currently unclear whether the absence of fish bones at many northern sites owes to a preservation issue, a methodological issue, or the actual insignificance of these resources in economic systems.

**B. Salmonids in Finnish Archaeology**

Prehistoric salmon fishing has not been adequately studied in Finland (^40^, and references therein). Bones are poorly preserved in acidic (~4-5) soils and sediments, and it is thought that diagenesis in these contexts increases bone fragmentation and brittleness, making bone specimens difficult to recover. These factors hinder the taxonomic study of prehistoric fish remains^41-44^. All fish bones from Stone Age contexts in Finland appear burned. They are very fragmentary, small, and the majority have presumably decomposed during the burning process^43^. The burned fish remains represent mostly fresh-water species, such as pike (*Esox lucius*), perch (*Perca fluviatilis*), pikeperch (*Sander lucioperca*), burbot (*Lota lota*), whitefish (*Coregonus lavaretus*), and cyprinid fish (e.g., roach (*Rutilus rutilus*), bream (*Abramis brama*)^43^. Few marine and migratory species (e.g. cod and salmon) are present in prehistoric fish bone assemblages. A total number of only ca. 1,100 salmonid remains have been recovered from the whole of Finnish archaeological sites and only six of these fragments have been identified as Atlantic salmon (*Salmo salar*)^40^.

The small number of salmon remains represented in the prehistoric archaeological record of Finland is striking, because during the historic period, rivers situated at the upper end of the Gulf of Bothnia, northwest Finland were known to have directed hundreds of thousands of migratory salmon into the Baltic Sea every year^44^. As a result, the prehistoric origin of salmon fishing has typically been perceived as a self‐evident fact in Finnish archaeology, even though direct osteological and artifactual evidence confirming this is scarce. The incredibly low frequencies of salmon remains in Finnish archaeofaunal assemblages have recently been explained as reflecting not only poor preservation and coarse excavation techniques but also processing, storage, and waste disposal which may be key factors affecting the representation of salmonid remains at archaeological sites^45^.

Contrary to the previous suggestions for Mid-Holocene communities of the Iijoki River in northwest Finland, who primarily based their subsistence strategies on sealing, it has recently been suggested that estuary based populations focused on mass-harvesting of anadromous fish ca. 3,500-3,000 cal BCE^40, 45^. Even though the direct osteological evidence confirming this is weak, site locations, fishing technology, and economic specialization may be assumed to have been associated with mass-harvesting and/or processing locations of migratory fish. Direct evidence for fish as a food source, especially in the case of protein-rich, seasonally abundant, and tasty salmonids, at Iijoki River sites is scarce.

**C. Study Sites in Finland**

**Site**: Yli-Ii (Kuivaniemi) Veskankangas

**Coordinates**: (ETRS89/WGS84), 65° 42' N, 25° 45' E

**Dating**: ca. 5,250-4,850 cal BCE^47, 48^

**Description**: The Stone Age settlement site of Kuivaniemi Veskankangas is located on the southern bank of the Kuivajoki River in northern Ostrobothnia, northwestern Finland^49^. The site lies on a plateau by the riverbank, its core area being approximately 50 by 200 m in size. Archaeological excavations have been in conducted during the 1950s, 80s, and 90s. The site has yielded an extensive collection of Late Mesolithic and Early (Sub-) Neolithic materials typical for northern Ostrobothnian sites, such as quartz, slate and quartzite, early pottery (Early Comb Ware) and burned bones. Thick cultural layers have been well preserved across a relatively undisturbed landscape containing several Stone Age structural remains and features such as hearths and postholes. During its occupation, the site was located on the estuary of the Kuivajoki River, but due to the rapid isostatic land uplift typical for this part of the country, it now lies several km from the current coastline.

**Bone samples used in this study:**

KM 24423:24 (Phocidae, tibia dex. dist. fr.)

KM 24423:37 (Phocidae, phal. fr.)

KM 24423:112 (Phocidae, phal. 1 prox. fr.)

KM 24423:293 (Phocidae, femur sin. dist. fr.)

KM 24928:986 (cf. *Salmo salar*, vertebra fr. 1)

KM 24928:1534 (Phocidae, calcaneum sin. fr.)

KM 24928:1544 (Phocidae, phal. 1 prox. fr.)

KM 24928:1560 (Phocidae, humerus dex. dist. fr.)

KM 24928:1561 (Phocidae, phal. 1/2 dist. fr.)

KM 24928:1609 (Phocida, enaviculare dex. fr.)

KM 25800:11 (Phocidae, phal. 1/2 dist. fr.)

**Site**: Nilsiä (Kuopio) Lohilahti

**Coordinates**: (ETRS89/WGS84), 63° 12' N, 28° 12' E

**Dating**: ca. 4,500-3,700 cal BCE^50, 51^

**Description**: The Lohilahti (*Fi.* Salmon Bay) site is located by the southern shore of Syväri Lake in northern Savo, Eastern Finland. Excavations to date are preliminary, but currently indicate that the lakeshore was occupied from ca. 4,500-3,700 cal BCE. Road building and sand extraction destroyed some parts of the roughly 200 by 100 m occupation area. The find materials include quartz, slate, ceramics (the early (Sub-) Neolithic pottery group (Early Asbestos Ware), and burned bones.

**Bone sample used in this study**:

KM 33378:124 (*Salmo salar*, vertebra fr. 1)

**Site**: Simo Tainiaro

**Coordinates**: (ETRS89/WGS84), 65° 51' N, 25° 29' E

**Dating**: ca. 5,000-4,700 cal BCE^50, 51^

**Description**: The Tainiaro site is located near the southern bank of the Simojoki River in southwest Lapland, northern Finland. Fieldwork at this extensive Early (Sub-) Neolithic site began in the 1960s and continued during the 1980s and 90s^49^. As result of several years of excavation, large amounts of archaeological finds have been collected, including slate, quartz, and flint artefacts, as well as sherds of Early Comb Ware pottery. The majority of burned bones are from seal and wild reindeer. Several structural remains have also been observed, such as hearths, pit features, and - interestingly - a number red-ochre inhumation graves. Tainiaro is an important Early Comb Ware site both nationally and internationally because of its rich combination of settlement and burial areas and associated archaeological materials.

**Bone samples used in this study**:

KM 25797:191 (*Rangifer tarandus*, phal. (tarsi) (prox. fr. 1)

KM 25797:195 (*Rangifer tarandus*, phal. (prox. fr. 1)

KM 26698:1163 (*Rangifer tarandus*, phal. (1 dist. fr. 1)

KM 26698:1179 (*Rangifer tarandus*, phal. (2 dist. fr. 1)

KM 26698:1224 (*Rangifer tarandus*, phal. (1 dist. fr. 1)

**Site**: Yli-Ii (Oulu) Kierikinkangas

**Coordinates** (ETRS89/WGS84), 65° 22' N, 25° 57' E

**Dating**: ca. 3,800-3,300 cal BCE^49^

**Description**: The settlement site of Kierikinkangas is located on the northern bank of the Iijoki River in northern Ostrobothnia, northwest Finland. This large site complex is constituted of at least five occupation areas, some parts of which have been destroyed due to sand extraction. Archaeological excavations began in the 1960s and projects have been in progress from the 1980s until today. At least 70 pit-house features and other structural remains have been recorded at the site. Some of the pit-house features are clustered in pairs and in row formations. The Kierikki Stone Age Centre was launched in the vicinity of the site in the early 2000s, and the Centre has organized public excavations since 2006. Kierikinkangas was an extensive Stone Age village by the riverbank during the Typical Comb Ware period (ca. 3,800-3,300 cal BCE). Apart from areas that have been excavated or destroyed by sand extraction, the site is still mostly intact and has high archaeological research potential. A large collection of archaeological finds have been recovered over decades of fieldwork, containing quartz, quartzite, flint, slate, and amber artefacts, pottery (mostly Typical Comb Ware style and its asbestos-tempered variants), and burned bones. Seal dominates in the faunal remains, as is the case with the other Kierikki sites.

**Bone samples used in this study**:

KM 31072:232 (Phocidae, mandibulasin. fr. 1)

KM 31072:543 (Phocidae, cranium, cond. occ. dex. fr. 1)

KM 31072:654 (Phocidae, cranium, meat. aud. ext. sin. fr. 1)

KM 31072:710 (Phocidae, cranium, occ. sin. fr. 1)

KM 31072:443 (Phocidae, costa fr.)

KM 31072:559 (Phocidae, costa fr.)

KM 31072:560 (Phocidae, axis fr.)

KM 31072:571 (Phocidae, phal. 1diaph. fr.)

KM 31072:575 (Phociae, cranium, cond. occ. dex. fr.)

KM 31072:586 (Phocide, phal. 1 dist. fr.)

KM 31072:590 (Phocidae, phal. 1 diaph. fr.)

KM 31072:596 (Phocidae, phal. 2 prox. fr.)

KM 31072:601 (Phocidae, costa fr.)

KM 31072:603 (Phocidae, costa fr.)

**Site**: Yli-Ii (Oulu) Kuuselankangas

**Coordinates**: (ETRS89/WGS84), 65° 22' N, 25° 56' E

**Dating**: ca. 3,600-3,100 cal BCE^49^

**Description**: The extensive Kuuselankangas site is located near the northern bank of the Iijoki River in northern Ostrobothnia, northwest Finland. The site is composed of approximately 30 pit-house features and some other types of pit structures, most of which are located in row formations. Dozens of archaeological excavations have been conducted at the site since the early 1990s^51-53^. The most typical find groups are quartz, quartzite, burned bones (mostly seal), potsherds (of Typical Comb Ware and asbestos-tempered Kierikki Ware), amber objects (beads, buttons and pendants), and flint points.

**Bone samples used in this study**:

KM 28943:602 (*Rangifer tarandus*, scapula sin./dex., margocervic. fr. 1)

KM 28943:607 (*Rangifer tarandus*, scapula sin., cavitas gleinoid. 1, scapula sin., margocervic. fr. 1)

KM 28943:661 (*Rangifer tarandus*, cornufr. 1)

KM 28943:716 (*Rangifer tarandus*, mt III-IV sin. prox. fr. 2)

**Site**: Yli-Ii (Oulu) Purkajasuo Korvala

**Coordinates**: (ETRS89/WGS84), 65° 22' N, 25° 54' E

**Dating**: ca. 3,400-2,900 cal BCE^53-55^

**Description**: The Stone Age settlement site of Purkajasuo Korvala is located near the northern shore of Iijoki River in northern Ostrobothnia, northwest Finland. During the Stone Age, the site was located in an estuary landscape by the mouth of the Iijoki River. Korvala is one part of an extensive site complex of Purkajasuo containing at least 60 pit-house features clustered into three groups. In the eastern and middle part of the site complex, the pit-houses form several row-house constructions. The lengths of the individual rooms vary between 18 and 29 m. The site has been excavated only partially in the late 1990s and early 2000s. This Mid to Late (Sub-) Neoltihic site has yielded an extensive collection of archaeological finds, e.g. quartz, flint, quartzite, polished stone tools, amber, early copper, pottery (Typical Comb Ware, asbestos-tempered Pöljä and Kierikki Wares), and remains of wooden log house structures. The contemporary fishery site of Purkajasuo, with well-preserved stationary wooden fishing structures, is situated on the northern side of Korvala^55^.

**Bone samples used in this study**:

KM 30602:273 (*Phoca groenlandica*, cranium, maedex. fr. 1)

KM 31020:1408 (*Phoca groenlandica*, cranium, maesin. fr. 1)

KM 31020:1475 (*Phoca groenlandica*, cranium, maesin. fr. 1)

KM 31020:1545 (Phocidae, cranium, praemax. sin .ant. fr.*)

KM 31020:1552 (Phocidae, phal. 2)

KM 31020:1560 (*Salmo salar* vertebra fr. 1)

KM 31020:1567 (Phocidae, praemaxillaresi. + dex. fr.)

KM 31020:1575 (Phocidae, cranium, jugale dex. fr.)

KM 31020:1576 (Phocidae, phal. 2 prox. fr.)

KM 31835:943 (*Phoca groenlandica*, cranium, maedex. fr. 1)

KM31835:990 (*Phoca groenlandica*, cranium, maesin. fr. 1)

KM 31835:1554 (*Phoca hispida*/*Phoca groenlandica*, cranium, maedex. fr. 1)

**Supplementary Information 2: Extended results**

**A: Mineralogical Phase Transformation in Atlantic Salmon Bone**

In a previous study, two of us (D.H.B. and R.S.-G.) observed the formation of whitlockite (WH: Ca_9_Mg (HPO_4_)(PO_4_)_6_) at 400 ^°^C and beta magnesium tricalcium phosphate (βMgTCP: Ca_8_Mg (PO_4_)_6_) at 600 ^°^C in sequentially heated fresh salmonid bones^56^. Both minerals can form by calcining Ca^2+^ deficient, Mg^2+^ rich hydroxylapatite (HAp: Ca_10_(PO_4_)_6_(OH)_2_)^56-63^. We documented the process of this mineralogical transformation using FTIR spectroscopy and XRD, and focus here on the FTIR indicators. The mineralogical transformation starts with decomposition of bone mineral carbonate (i.e., decarbonation) which was identified in infrared spectra by the loss of the 875 cm^−1^ carbonate (CO_3_) *v*3 absorbance band. The small band at 633 cm^−1^ indicates the presence of a small amount hydroxyl (OH)^64, 65^. Hydrogen phosphate (HPO_4_) was also altered, deduced from the characteristic band at 880 cm^−1^. Both CO_3_ and HPO_4_ bands were completely lost in salmonid bones burned between 600 ^°^C and 700 ^°^C.

We identified the formation of WH at 400 ^°^C, which was indicated by the appearance of an infrared absorbance shoulder at 1,150 cm^−1^. This is typical of bone with Ca^2+^ deficiencies^56, 57^. Indeed, we documented Ca/P ratios <1.67 in our experimental samples using XRD. Ca^2+^ deficiency is expected in Atlantic salmon that lose a fraction of their Ca^2+^ stores during spawning because they stop feeding during this freshwater stage of their life cycle^58, 63^. As a result, the skeleton looses Ca^2+^ and takes on a more cartilaginous form^58^.

With increased heating temperature we found that WH was replaced by βMgTCP at 600 ^°^C. Infrared spectra showed losses of the WH shoulder at 1,150 cm^−1^ and CO_3_/HPO_4_ bands at 875-880 cm^−1^, along with the formation of several βMgTCP bands, such as 1,120 cm^−1^ and 985 cm^−1^. WH is known to transform into βMgTCP when HPO_4_ is decomposed, the loss of which, as mentioned, was confirmed in the infrared analyses^59^. This mineral has characteristic bands at 1,015 cm^−1^, 985 cm^−1^, 613 cm^−1^, and 555 cm^−1^ ^59^. The intensification of βMgTCP infrared absorbance bands at temperatures above 600 ^°^C signified the continued formation of βMgTCP in our previous experiments.

Overall, we have shown that WH and βMgTCP form in salmonid bones, in relation to Ca^2+^ deficiencies. We provided a set of criteria by which these minerals can be identified in burned salmonids, and proposed that this can be used to study archaeological burned salmonid remains. In the current study we first repeated the experiments with fresh bones and then used these criteria to study archaeological samples from a set of subarctic sites in Finland.

**B. Determination of Heating Temperature in Atlantic Salmon Bone**

Extensive research has been conducted on the use of FTIR spectroscopy to estimate bone burning temperatures as reflected in increased bone mineral crystallinity^64-76^. The infrared splitting factor (IRSF) is the most widely used measure of bone crystallinity. This value is calculated by dividing the sum of the 605 cm^-1^ and 565 cm^-1^ band heights by the height of the valley splitting them^74^. Burning improves atomic order in the HAp crystal lattice. It increases the sizes of HAp crystals eventually causing them to sinter and agglutinate, making the mineral more crystalline, in turn increasing the IRSF. For example, values for modern unaltered mammal bone typically have IRSF values between 2.5 and 3. Burned bones have the following IRSF values: 200 ^°^C - 500 ^°^C = IRSF 3.1 - 3.9; 600 ^°^C - 700 ^°^C = IRSF 4 - 5; 800 ^°^C = IRSF > 5 ^70-73^.

It has been argued, however, that using the IRSF in isolation fails to provide accurate estimations of firing temperatures because bone crystallinity changes in relation to two variables, heat intensity and heat duration. Archaeological burned bone crystallinity also depends on post-depositional changes^76^. Therefore, determination of past heat exerted on bone is often supplemented with measurements of CO_3_, carbonyl (C-O), and OH, as well as changes in the PO_4_ *v*3 band. Carbonate, for example, begins to decompose at roughly 600 ^°^C and is typically fully decomposed at around 800 ^°^C, making it a useful estimator of heating between these temperatures^74^. Changes in CO_3_ can be tracked using the CO_3_ *ν*2 to PO_4_ *v*3 ratio. Unaltered mammal bone has been shown to have a ratio between 0.65 and 0.31, while values lower than 0.04 indicate burning at temperatures surpassing 700 ^°^C^70^. Burning at temperatures over 600 ^°^C can also be estimated using the formation of OH. Beginning at 500 ^°^C to 600 ^°^C, the OH absorbance band begins to form on the left side of the PO_4_ *v*4 at 633 cm^−1^, and it gradually becomes more pronounced with increasing temperature^70^. The OH forms *via* the interaction of CO_3_ thermal decomposition products and remaining structural water or HPO_4_ ^65^. The formation of OH has been tracked using the ratio of its band at 633 cm^−1^ to the 605 cm^−1^ PO_4_ *v*4 band^70^. At 600 ^°^C an additional sharp band representing free OH forms at 3,575 cm^−1 77^. A sharper, narrower, left-shifted PO_4_ *v*3 band with a deep split at 1,090 cm^−1^ has also been used to identify highly burned bone^74^.

Using our experimental model of how these components were influenced during the burning process of modern Atlantic salmon bone, we can estimate the temperatures to which archaeological salmon bones have been exposed. Results are presented in Supplementary Table 1. The Nilsiä Lohilahti (NL) and Yli-Ii Purkajasuo Korvala (YP) samples showed signs of burning at temperatures of 600 ^°^C to 700 ^°^C. The decomposition of the organic components, demonstrated by the loss of C-O in the spectra, indicated burning at temperatures over 400 ^°^C. The presence of the OH band at 3,575 cm^−1^ and neoformed βMgTCP confirmed heating at 600 ^°^C or higher. It must be noted that the βMgTCP bands in the archaeological specimens were far less pronounced than those formed in the experimental samples 600 ^°^C, a result of mineral hydration in the sedimentary context after burning. Values for the CO_3_ *v*3/PO_4_ *v*3 ratio were similar to those of experimental samples burned at 600 ^°^C to 700 ^°^C.

The Kuivaniemi Veskankangas (KV) sample was burned at 800 ^°^C. The presence of WH in the sample gives the appearance that the bone was burned at temperatures of 400 ^°^C to 500 ^°^C. Yet, the presence of the OH band at 3,575 cm^−1^ and the low CO_3_ *v*3/PO_4_ *v*3 values suggested firing at temperatures greater than 600 ^°^C. Moreover, the results of our hydration experiments demonstrated that the large amount of βMgTCP formed at high temperatures can transform into WH when exposed to acidic solution. It is therefore reasonable that the WH formed from hydrated βMgTCP initially produced at 800 ^°^C. The IRSF is quite high, at 6.88, likely indicating the transformation of a large amount of βMgTCP into highly crystalline HAp. It is unlikely that the bone was burned at temperatures beyond 800 ^°^C, as open wood fuelled hunter-gatherer fires are not know to sustain temperatures this high for long periods of time^78-80^.

Our experiments also showed that crystalline HAp forms when burned Atlantic salmon bones are hydrated. This raises several methodological issues. (1) Measurements of HAp crystallinity can not be used to assess burning temperatures in Atlantic salmon bone. When βMgTCP has formed, and has remained largely unaltered, its absorbance bands distort the PO_4_ *v*3 and *v*4 bands, thus making accurate measurements of HAp crystallinity impossible. The issue is particularly pronounced in the IRSF measurement. IRSF values for modern bone were lower than expected at temperatures of 600 ^°^C and higher, which owes primarily to the shallower split between the 605 cm^−1^ and 565 cm^−1^ PO_4_ *v*4 bands. This is a result of the developing 590 cm^−1^ βMgTCP band. As demonstrated, hydrating burned salmon bone also increases the crystallinity of HAp, therefore, bone burned at temperatures above 600 ^°^C that has been hydrated is expected to have a higher IRSF, which is misleading in the context of temperature estimation. For example, the modern bone burned at 600 ^°^C produced an IRSF of 3.74, while the two archaeological samples (NK and YP) suspected to have been burned at 600 ^°^C to 700 ^°^C have values over 5. Note that the IRSF can be calculated for the archaeological samples containing βMgTCP because hydration has reduced the quantity of βMgTCP, which removed its influence on the PO_4_ *v*4 band.

(2) Using the OH/PO_4_ *v*4 605 cm^−1^ ratio to estimate the heating temperature of burned Atlantic salmon bone is also problematic. This is ultimately a result of the lower amounts of HPO_4_ initially present in the samples. As mentioned, this component is involved in the formation of the OH band at 633 cm^−1^ at temperatures of 600 ^°^C or higher. Our experiments showed that Atlantic salmon bone lost HPO_4_ earlier in the firing sequence relative to the reindeer and seal bones thus limiting its availability for reaction with CO_3_ decomposition products. This explains the small amount of OH formed in burned salmon bone. The 605 cm^−1^ of the PO_4_ *v*4 is also increased when βMgTCP is present in large quantities. In a very well preserved, unaltered specimen the poor formation of the 633 cm^−1^ and more pronounced 605 cm^−1^ would provide a misleading temperature estimation. Moreover, our hydration experiments showed that the OH band becomes increasingly more pronounced with continued hydration. The archaeological salmon samples differ from the experimental samples in terms of their OH and PO_4_ *v*4 components. Values for the OH/PO_4_ *v*4 605 cm^−1^ ratio in the archaeological samples far surpass those for the modern samples burned at 1,000 ^°^C, suggesting that the samples were burned at temperatures beyond 1,000 ^°^C, which is unlikely to occur for any sustained period of time in an open wood fuelled hunter-gatherer hearth^78-80^.

(3) Our results showed that the OH band at 633 cm^−1^ does not exclusively derive from burning. It may also develop from hydration. This band, or indices including it, should not be used in isolation when estimating bone burning temperatures. We expect that hydration in the archaeological depositional environment will influence the OH component of other animal bones, thereby compromising the accuracy of the OH/PO_4_ *v*4 605 cm^−1^ in bone burning temperature estimations. Importantly, this suggests that the ratio should be used cautiously, or not at all, when attempting to determine burning temperatures in any type of archaeological bone specimen that has been retrieved from a wet context, as these bones are expected to have experienced some degree of hydration after they were burned. This proposition requires further investigation.

**C. Yli-Ii Kierikinkangas Hearth**

At least 70 pit-house features have been identified at the Yli-Ii Kierikinkangas archaeological site since its discovery in 1960. Excavations of several houses demonstrated that open wood-fuelled hearths were placed in their centres. We investigated one of these features in June/July of 2017. The soil in the study area is an acidic podzol, indicated by a thick eluviated, silicate rich horizon followed by an illuviated, iron rich horizon. Fluvial sands dominated by quartz and feldspar serve as the parent materials for the soil in this study area. These sands are well rounded and range from fine to coarse in texture.

A total of 1,166 bone fragments were recovered by fine sieving of the bulk samples. Of these bones, 3.9 % could be identified to the family or species level. The identified fragments represent typical species for the Iijoki riverbank sites: Phocidae and mesomammalia (n=14) and fish (n=31) (pike, whitefish, cyprinids, and Teleostei).

As mentioned in the main text, 136 particles were selected for FTIR characterization. Thirty of these had spectra characteristics similar to WH, and 12 of these 30 had characteristics clearly derived from HAp and WH. WH was identified by the presence of bands at 1,150 cm^−1^, 1,120 cm^−1^, and 1,070 cm^−1^ in the PO_4_ *v*3 region, and bands at 550 cm^−1^ and 575 cm^−1^, in the PO_4_ *v*3 region. WH was distinguishable from βMgTCP in these cases by the presence of the 1,150 cm^−1^ band and the HPO_4_ band at 880 cm^−1 56, 57^. Archaeological bones from Finnish archaeological contexts, including Yli-Ii Kierikinkangas, did not contain these mineral phases. Comparisons with offsite sediments also indicated that the WH peaks were unique to the fragments recovered from the archaeological hearth. Absorbance bands characteristic of WH were not identified in any other component of the hearth mineral assemblage, which included HAp, hematite, quartz, orthoclase feldspar, and oligoclase feldspar.

Most fragments containing WH were black. This likely owes to two factors. First, the fragments were taken from a charcoal rich context. Charred organics were identified in the FTIR spectra of the WH containing fragments, specifically by sloping baselines from 4,000 cm^−1^ to 1,900 cm^−1^, large shoulders in the 900 cm^−1^ to 1,000 cm^−1^ region, and the hump-like appearance of the PO_4_ *v*4 region. These features were also identified in the spectra from archaeological charcoal samples. Second, our hydration experiments demonstrated the blackening of samples hydrated with HCl. As discussed above, the WH containing fragments retrieved from the hearth context showed signs that they were derived from salmon bones burned at temperatures exceeding 500 ^°^C. Our burning experiments revealed that βMgTCP forms in Atlantic salmon bone heated at 600 ^°^C and higher, and our hydration experiments demonstrated that βMgTCP produced at 800 ^°^C and subsequently hydrated with HCl can reform WH. Thus, the blackening of the bone fragments and the evidence for high temperature firing indicated that WH was formed *via* the hydration of βMgTCP in acidic hearth sediments.

The pH of the hearth sediment was 6.94. This was far less acidic than the values recorded for the archaeological sediment outside the hearth area (pH=4.19) and the sediment collected off-site (pH=3.31). The neutral pH of the hearth sediment is likely due to incorporation of alkaline wood-ash and burned bone powders into the hearth sediment matrix^81^. The occurrence of burned bone and alkaline wood ash induces local alkaline conditions that will protect bone mineral from complete dissolution despite the acidic nature of the surrounding soils, thereby preserving a fraction of the deposited materials. This proposition is supported by studies on the preservation of archaeological remains in prehistoric caves where acidic conditions develop under degrading bat guano^82^.

**D. Influences of Hydration on Burned Atlantic Salmon Bone Having Different Amounts of βMgTCP**

Our hydration experiments on modern burned fish bone provided insight into the archaeological preservation potential of WH and βMgTCP. To reiterate, sets of samples burned at 600 ^°^C, 800 ^°^C, and 1,000 ^°^C were hydrated with distilled H_2_O, NaClO, and HCl. Changes in mineralogy were tracked using FTIR. H_2_O hydration of bone burned at 600 ^°^C showed that βMgTCP bands were gradually reduced throughout the treatment process. More importantly, however, certain bands were rapidly diminished, while others were more resilient. The 555 cm^−1^ band of βMgTCP was lost after the first treatment, while the 1,075 cm^−1^, 947 cm^−1^, 590 cm^−1^, and 613 cm^−1^ bands were lost after the second treatment. After four treatments, the 1,120 cm^−1^ and 985 cm^−1^ were the only identifiable βMgTCP bands present. Larger quantities of βMgTCP were produced at 800 ^°^C and 1,000 ^°^C, which had a profound impact on band reduction during the H_2_O hydration process. After four treatments, the bone burned at 800 ^°^C lost the 1075 cm^−1^, 613 cm^−1^, and 555 cm^−1^ bands. The rest of the bands were very well resolved. All of the βMgTCP bands in the sample burned at 1,000 ^°^C were near perfect at the end of the hydration experiment. These results indicated that when βMgTCP is present in large quantities, it is more resistant to changes caused by hydration with H_2_O.

Small changes in βMgTCP absorbance bands were observed in the NaClO experiments, suggesting that βMgTCP should not experience a high degree of transformation in alkaline sediments. As expected, the HCl treatment process greatly affected the βMgTCP bands. Similar to the H_2_O experiments, however, the 1,120 cm^−1^ and 985 cm^−1^ bands were the most resilient, identifiable in most of the treated samples. This suggested that these bands are likely the most useful traits for identifying βMgTCP in archaeological bones excavated from acidic sediments. The larger amounts of βMgTCP produced at 800 ^°^C and 1,000 ^°^C were less affected than those in the samples burned at 600 ^°^C, again suggesting that the extent of βMgTCP transformation is related to the amount initially produced. In addition, it appears that the introduction of acidic solution caused some of the βMgTCP to hydrolyze into WH.

**Supplementary Figures**

**Figure S1: Infrared spectra of archaeological bones.** The phosphate *v*3 and *v*4 bands for bones of seal (**A**), reindeer (**B**), fish (**C**), and unidentifiable species (**D**) collected from different archaeological sites in Finland. Sites and catalogue numbers are provided where available. Wavenumbers (cm^−1^) for each absorbance band are specified. Abbreviations: PO_4_ = phosphate; HAp = hydroxylapatite; HPO_4_ = hydrogen phosphate; CO_3_ = carbonate; OH = hydroxyl.

**
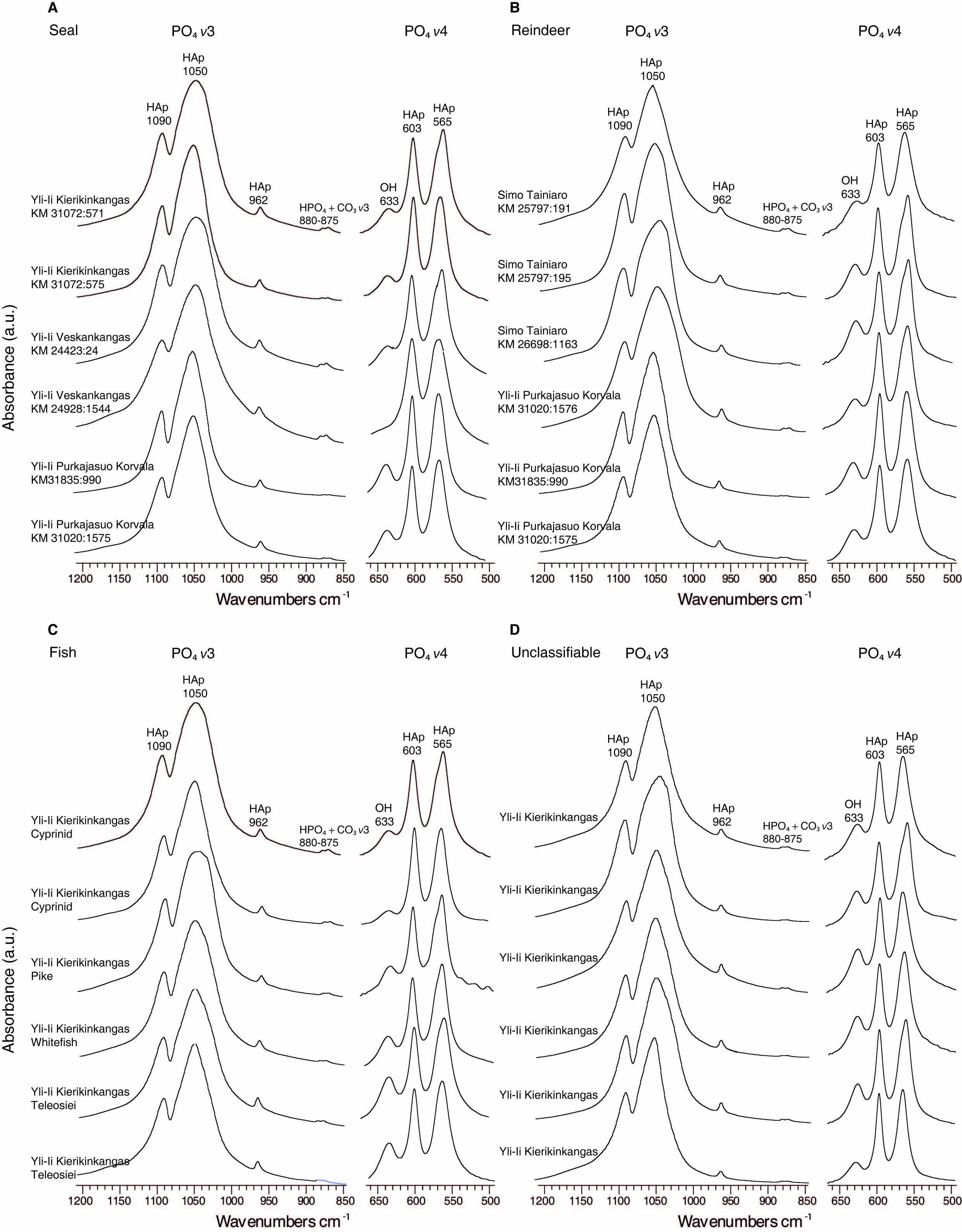
**

**Figure S2: The hearth sampling context at the Yli-Ii Kierikinkangas archaeological site**. (**A**) The Yli-Ii Kierikinkangas site plan showing the location of the pit-house containing the sampled hearth. (**B**) A photograph of the excavated pit-house showing the hearth sampling location. (**C**) An example of sediment sampling locations outside of the hearth context. (**D**) An example of sampling locations inside the hearth feature. The location of sediment sample L6-2 is highlighted where several of the fragments exhibiting infrared absorbance bands for whitlockite have been identified. Map by Kierikki Stone Age Centre/Sami Viljanmaa.


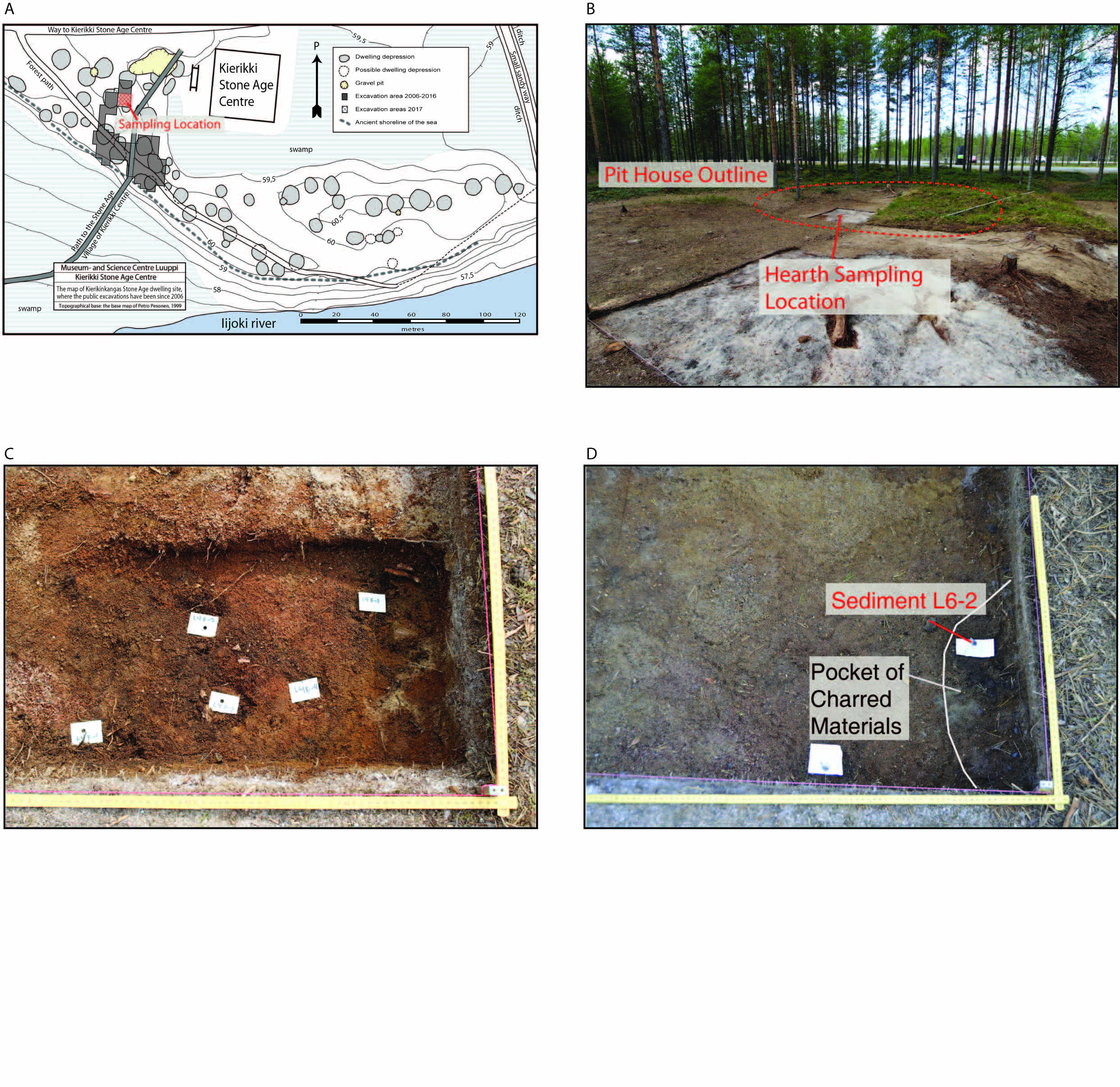


**Figure S3: Photographs showing modern and archaeological salmonid bones.** The results of hydration experiments showing the darkening of Atlantic salmon bone after hydration (**A**, **B**) and archaeological bone specimens without (**C**) and with (**D**) the whitlockite mineral.


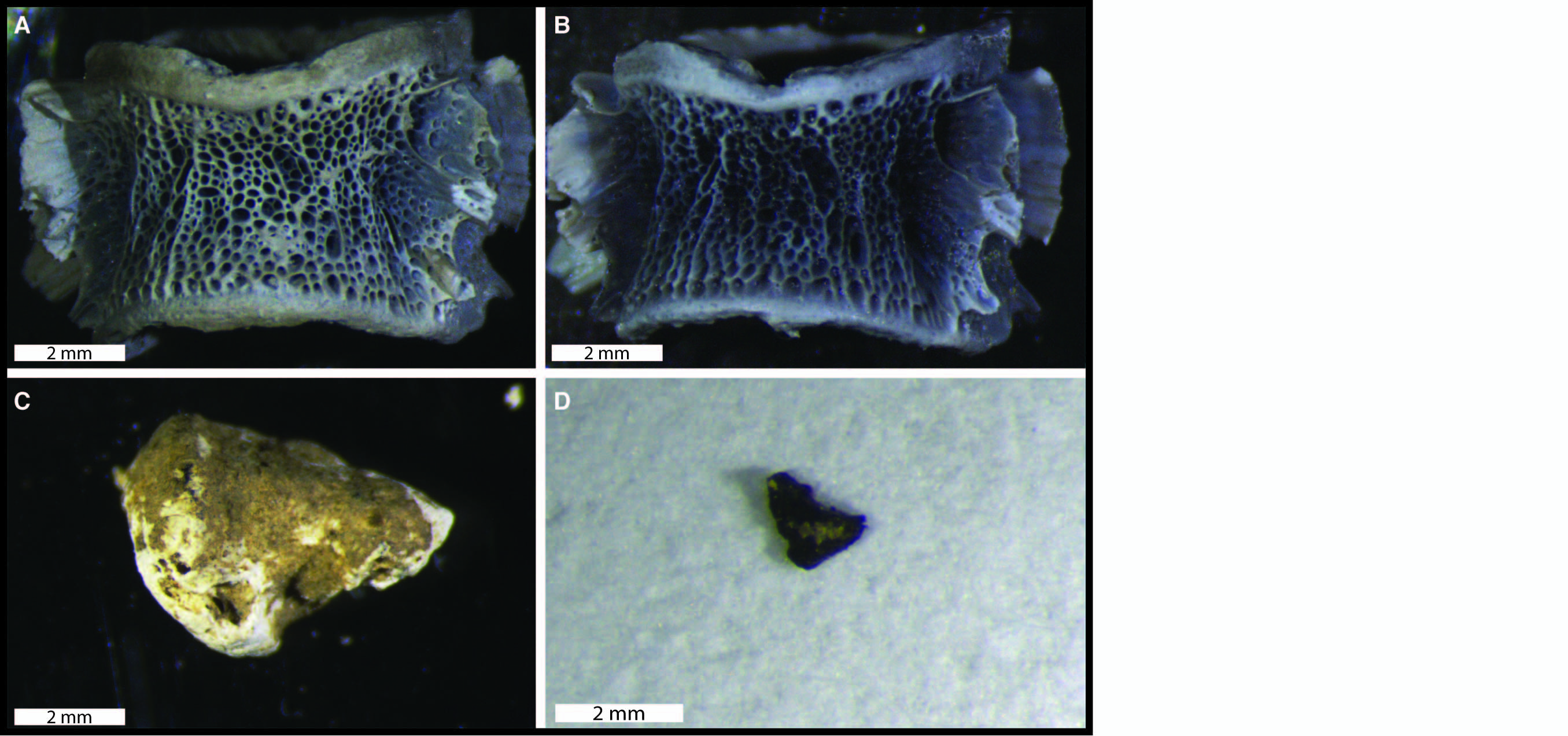


**Figure S4: Comparisons of infrared spectra for whitlockite with additional sediment components of the hearth sampling context at the Yli-Ii Kierikinkangas archaeological site.** Wavenumbers (cm^−1^) for each diagnostic absorbance band are specified. Bands for whitlockite and hydroxylapatite are labelled in red and black respectively. Abbreviations: HAp = hydroxylapatite; WH = whitlockite.

**
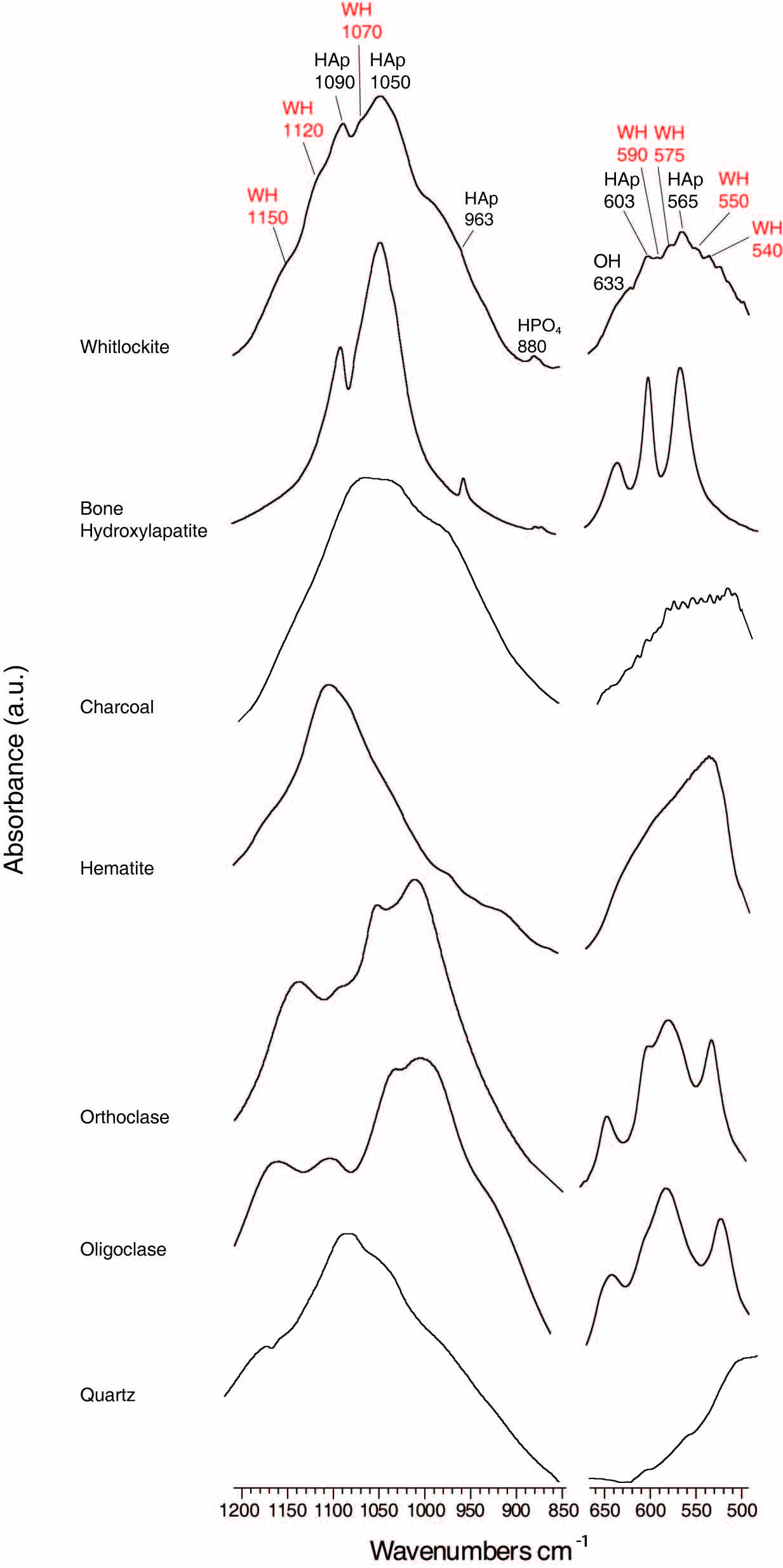
**

**Figure S5: Additional results for the modern burned fish bone hydration experiments.** Infrared spectra showing *v*3 and *v*4 phosphate absorbance bands for modern northern pike (**A**), bream (**B**), and cod (**C**) burned at 800 ^°^C. The uppermost spectra in each panel are the experiment starting points of burned but not hydrated bone, while the lower spectra are the results of 50 µl treatments with the designated solution. Wavenumbers (cm^−1^) for each absorbance band are specified, and bands for beta magnesium tricalcium phosphate are labelled in red. Abbreviations: PO_4_ = phosphate; HAp = hydroxylapatite; OH = hydroxyl; TCP = beta magnesium tricalcium phosphate; H_2_O = water; HCl = hydrochloric acid.


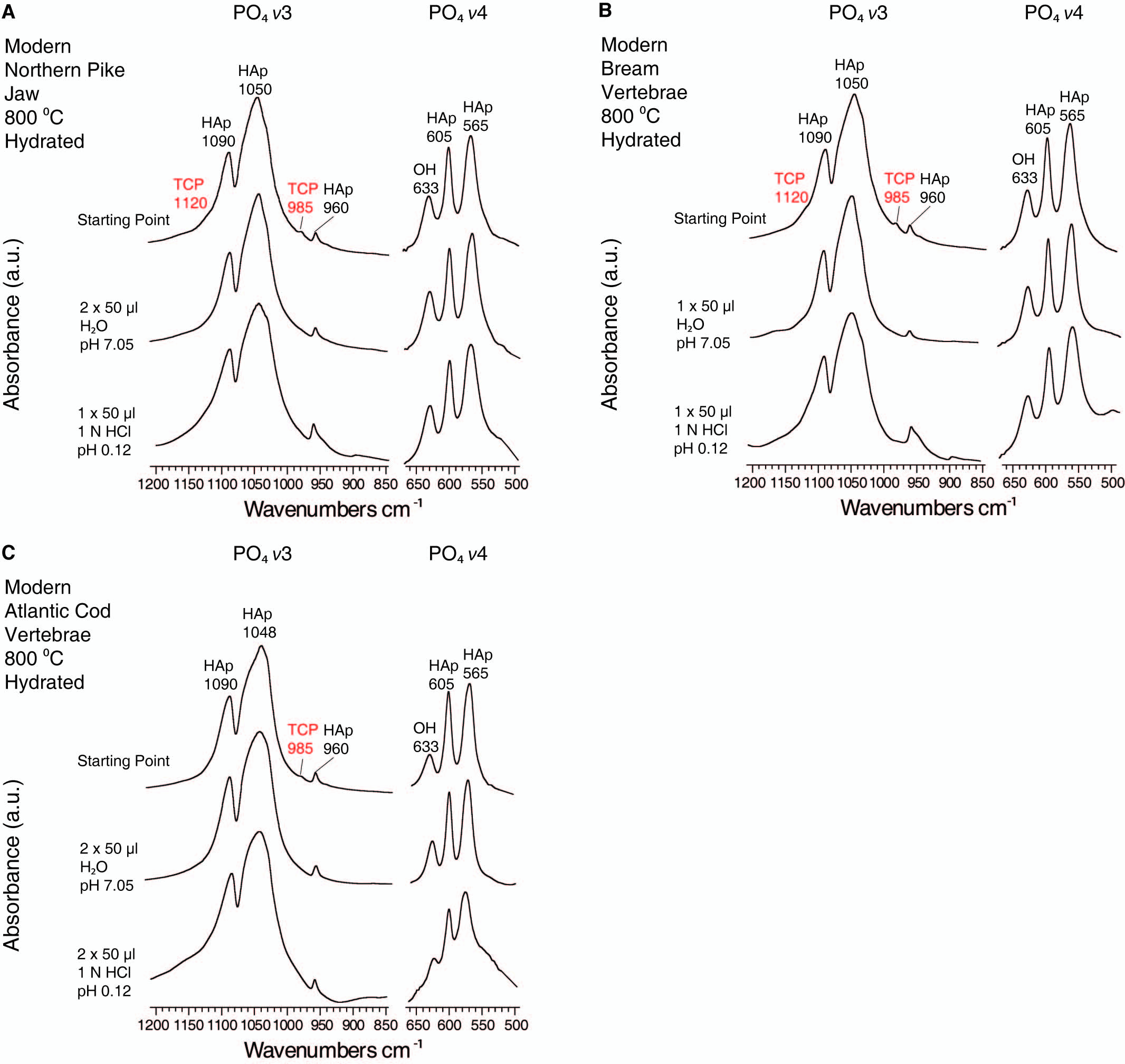


**Figure S6: MicroCT images of Atlantic salmon bone.** (**A**) Modern air dried bone. (**B**) modern bone burned at 800 ^°^C. (**C**) Modern bone burned at 800 ^°^C then hydrated with distilled water. (**D**) Archaeological bone from the Nilsiä Lohilahti site. The tissue mineral density (TMD) is displayed. Note the increase in TMD from fresh to burned to hydrated bone and the significantly high TMD in the archaeological bone. Note also that hydration in the modern burned bone (**C**) appears to form concentric rings that are clearly visible in the archaeological specimen (**D**).

**
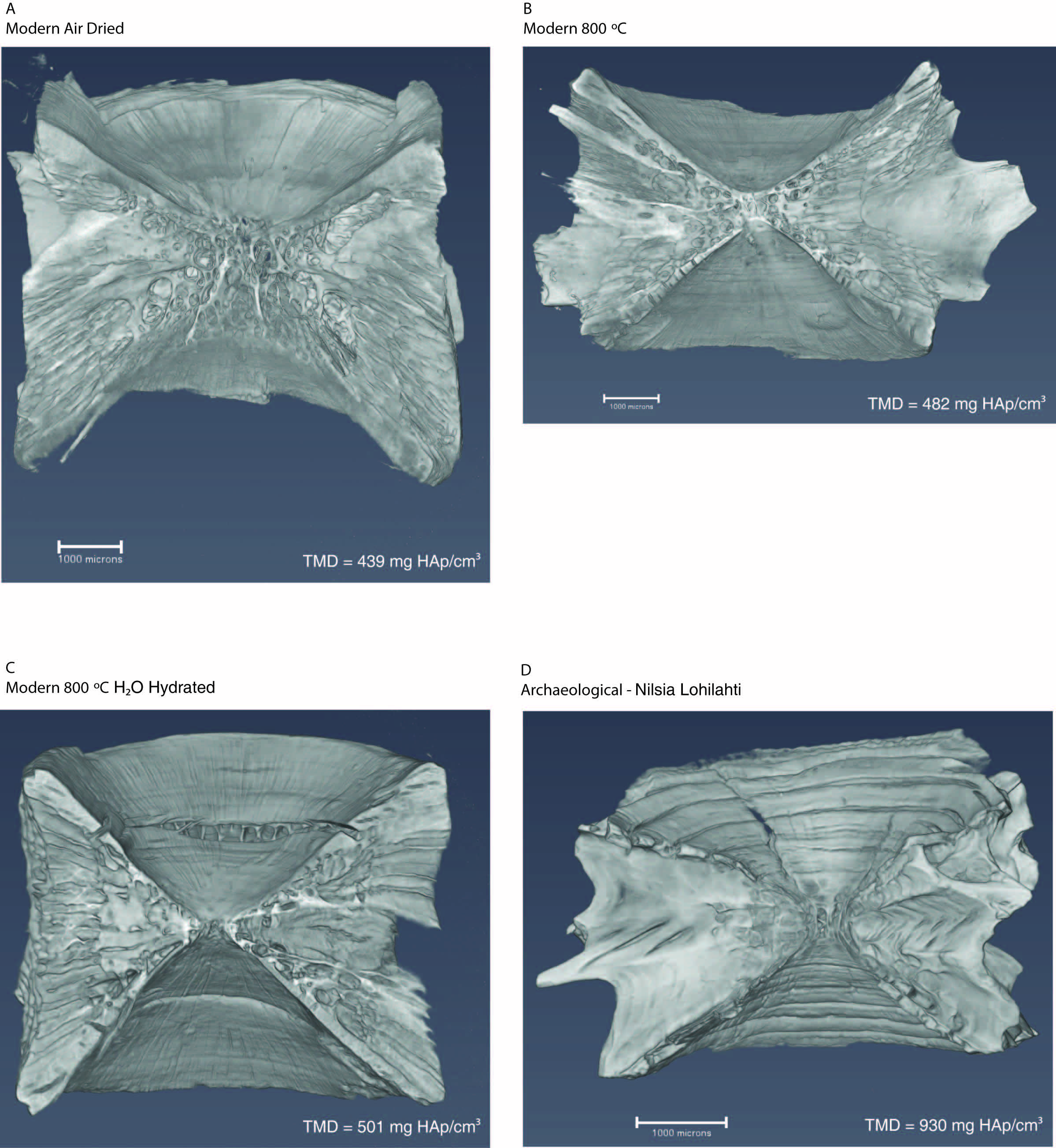
**

**Supplementary Table S1: Infrared spectroscopy assessments of bone burning temperatures for Atlantic salmon.** Results for the modern Atlantic salmon bone burned at temperatures between 100 ^°^C and 1,000 ^°^C are listed first, followed by the results for archaeological samples. Abbreviations: PO_4_ = phosphate; C-O = carbonyl; CO_3_ = carbonate; OH = hydroxyl; IRSF = infrared splitting factor; βMgTCP = beta magnesium tricalcium phosphate; WH = whitlockite; NL = Nilsiä Lohilahti; YP = Yli-Ii Purkajasuo Korvala; KV = Kuivaniemi Veskankangas.

| **Temp ^°^C** | **IRSF**  **±0.1** | **CO_3_ *v*3 /PO_4_ *v*3** | **CO_3_ *v*2**  **/PO_4_ *v*3** | **C-O**  **/CO_3_ *v*3** | **C-O**  **/PO_4_ *v*3** | **OH @ 633 cm^−1^ / PO_4_ *v*4** | **OH @ 3575** **cm^−1^** | **Neoformed Minerals** | |
| --- | --- | --- | --- | --- | --- | --- | --- | --- | --- |
| 25 | 2.3 | 0.66 | 0.040 | 3.1 | 2.3 | - | - | - | |
| 100 | 2.6 | 0.26 | 0.014 | 3.5 | 0.8 | - | - | - | |
| 200 | 2.5 | 0.31 | 0.031 | 5.2 | 1.3 | - | - | - | |
| 300 | 3.2 | 0.15 | 0.014 | 2.1 | 0.2 | - | - | - | |
| 400 | 2.9 | 0.075 | 0.013 | 1.4 | 0.0 | - | - | WH | |
| 500 | 3.1 | 0.045 | 0.005 | 0 | 0 | - | - | WH | |
| 600 | 3.7 | 0.006 | 0 | 0 | 0 | 0.24 | 🗸 | βMgTCP | |
| 700 | 4.6 | 0.005 | 0 | 0 | 0 | 0.17 | 🗸 | βMgTCP | |
| 800 | 4.5 | 0.004 | 0 | 0 | 0 | 0.18 | 🗸 | βMgTCP | |
| 900 | 4.5 | 0.004 | 0 | 0 | 0 | 0.42 | 🗸 | βMgTCP | |
| 1000 | 4.6 | 0.002 | 0 | 0 | 0 | 0.22 | 🗸 | βMgTCP | |
| **Archaeological Samples** | | | | | | | | |  |
| NL | 5.6 | 0.007 | 0 | 0 | 0 | 0.51 | 🗸 | βMgTCP | |
| YP | 5.9 | 0.005 | 0 | 0 | 0 | 0.46 | 🗸 | βMgTCP | |
| KV | 6.8 | 0.007 | 0 | 0 | 0 | 0.47 | 🗸 | WH | |

**Supplementary References**

1. Halffman, C. M. *et al*. Early human use of anadromous salmon in North America at 11,500 y ago. *PNAS* **112**, 12344-12348 (2015).

2. Betts, M. Zooarchaeology and the reconstruction of ancient human-animal relationships in the Arctic. In *The Oxford Handbook of the Prehistoric Arctic* (eds Friesen, M. & Mason, O.) 81-108 (Oxford Univ. Press, 2016).

3. Yesner, D. R. Faunal extinction, hunter-gatherer foraging strategies, and subsistence

diversity among eastern Beringian Paleoindians. In *Foragers of the Terminal Pleistocene in North America* (eds Walker, R. B. & Driskell, B. N.) 15-31 (Univ. of Nebraska Press, 2007).

4. Potter, B. A., Holmes, C. E. & Yesner, D. R. Technology and economy among the earliest prehistoric foragers in interior eastern Beringia. In *Paleoamerican Odyssey. Proceedings of the 2013 Paleoamerican Odyssey Conference in Santa Fe, New* *Mexico* (eds Graf, K. E, Ketron, C. V. & Waters, M. R.) 83-103 (Texas A&M Press, 2013).

5. Helmer, J. W. The Paleo-Eskimo prehistory of the Northern Devon Lowlands. *Arctic* **44**, 301-317 (1991).

6. McGhee, R. Paleoeskimo occupations of Central and High Arctic Canada. In *Eastern Arctic Prehistory: Paleoeskimo Problems* (ed. Maxwell, M. S.) 15-39 (Society for American Archaeology, 1976).

7. Independence I https://natmus.dk/organisation/forskning-og-formidling/nyere-tid-og-verdens-kulturer/etnografisk-samling/arktisk-forskning/prehistory-of-greenland/independence-i/. (National Museum of Denmark, 2018).

8. Milne, S. B. & Donnelly, S. M. Going to the birds: examining the importance of avian resources to Pre-Dorset subsistence strategies on Southern Baffin Island. *Arct. Anthropol.* **41**, 90-112 (2012).

9. McAvoy, D. G. An examination of the Pre-Dorset caribou hunters from the deep interior of Southern Baffin Island, Nunavut, Canada. (Unpublished Masters Thesis, Univ. of Manitoba, 2014).

10. Murray, M. Economic change in the Palaeoeskimo prehistory of the Foxe Basin, NWT.

(Unpublished PhD dissertation, McMaster Univ., 1996).

11. Arnold, C. D. *The Lagoon Site (OjRl-3): Implications or Paleoeskimo Interactions*. (National Museum of Man, 1981).

12. Whitridge, P. Zen fish: a consideration of the discordance between artifactual and zooarchaeological indicators of Thule Inuit fish use. *J. Anth. Archaeol.* **20**, 3-72 (2001).

13. Murray, M. Local heroes. the long‐term effects of short‐term prosperity ‐ an example from the Canadian Arctic. *World Archaeol*. **30**, 466-483 (1999).

14. Taylor, W. E. Summary of archaeological field work on Banks and Victoria Islands, Arctic Canada, 1965. *Arct. Anthropol.* **4**, 221-243 (1967).

15. Milne, S. B., Park, R. W. & Stenton, D. R. Dorset culture land use strategies and the case of inland southern Baffin Island. *Can. J. Archaeol*. **36**, 267-288 (2012).

16. Todisco, D. & Monchot, H. Bone weathering in a periglacial environment: the Tayara site (KbFk-7), Qikirtaq Island, Nunavik (Canada). *Arctic* **61**, 87-101 (2008).

17. Gordon, B. C. *People of the Sunlight, People of the Starlight: Barrenland Archaeology in the Northwest Territories of Canada*. (Canadian Museum of Civilization, 1996).

18. Dawson, P. C. *et al.* A Report on 2009 Archaeological Excavations Carried out at JjKs–7, Maguse Lake, Nunavut. (Department of Culture, Language, Elders, and Youth, Government of Nunavut, Iqaluit, 2009).

19. Dawson, P. C. *et al*. A Report on Archaeological Fieldwork at Ikirahak, Maguse Lake, Nunavut. Permit Report 2008–023a. Department of Culture, Language, Elders, and Youth, Government of Nunavut, Iqaluit, 2008).

20. Dawson, P. C. *et al.* A Report of Archaeological Fieldwork Undertaken in 2007 at Kuuvik (Jiku–1; JiKu–2). Permit Report 07–004a. (Department of Culture, Language, Elders, and Youth, Government of Nunavut, Iqaluit, 2007).

21. Ukkonen, P. Pohjois-Suomen eläimistön historiaa [Abstract: Faunahistory in northern Finland]. In Varhain pohjoisessa - Early in the North, maa - the land (eds Schulz, E.-L. & Carpelan, C.) Helsinki Papers in Archaeology 10, 49-57. (University of Helsinki, 1997).

22. Björck, S. A review of the history of the Baltic Sea, 13.0-8.0 ka BP. *Quat. Int*. **27**, 19-40 (1995).

23. Ukkonen P (1997) Pohjois-Suomen eläimistön historiaa [Abstract: Faunahistory in northern Finland]. *Varhain pohjoisessa - Early in the North, maa - the land* (Helsinki Papers in Archaeology 10, eds Schulz, E-L, Carpelan C (University of Helsinki, Helsinki), pp. 49-57.

24. Nurminen, K. Kalanluulöytöjä Suomen neoliittisilta asuinpaikoilta. *Muinaistutkija* **1**, 2-17 (2007).

25. Enghoff, I. B., MacKenzie, B. R., & Nielsen, E. E. The Danish fish fauna during the warm Atlantic period (ca. 7000-3900 BC): Forerunner of future changes? *Fisheries Research* 87, 167-180 (2007).

26. Heikkilä, M. & Seppä, H. An 11,000 yr palaeo temperature reconstruction from the southern boreal zone in Finland. *Quat. Sci. Rev*. **22**, 541-54 (2003).

27. Zvelebil, M. Innovative hunter-gatherers: the Mesolithic in the Baltic. In *Mesolithic Europe* (eds Bailey, G. & Spikings, P.) 18-59 (Cambridge Univ. Press, 2008).

28. Bjerck, H. B. Norwegian Mesolithic trends: a review. In *Mesolithic Europe* (eds Bailey, G. & Spikings, P.) 60-106 (Cambridge Univ. Press, 2008).

29. Bergsvik, K. A., Hufthammer, A. K. & Ritchie, K. The emergence of sedentism in Mesolithic western Norway: a case-study from the rockshelters of Sævarhelleren and Olsteinhelleren by the Hardanger fjord. In *Marine Ventures-Archaeological Perspectives on Human-Sea Relations* (eds Bjerck, H. B. *et al*.) 33-51 (Equinox Publishing, 2016).

30. Hansson, A. *et al*. A submerged Mesolithic lagoonal landscape in the Baltic Sea, south-eastern Sweden - early Holocene environmental reconstruction and shore-level displacement based on a multiproxy approach. *Quat. Int*. **463**, 110-123 (2016).

31. Groß, D. *et al*. People, lakes and seashores: studies from the Baltic Sea basin and adjacent areas in the early and Mid-Holocene. *Quat. Sci. Rev.* **185**, 27-40 (2018).

32. Boethius, A. Something rotten in Scandinavia: the world's earliest evidence of fermentation. *J. Archaeol. Sci*. **66**, 169-180 (2016).

33. Boethius, A. Fishing for ways to thrive. Integrating zooarchaeology to understand subsistence strategies and their implications among Early and Middle Mesolithic southern Scandinavian foragers. *Acta Archaeologica Lundensia* (8)70. Studies in Osteology 4. Lund University (2018).

34. Ritchie, K. C. The Ertebølle Fisheries of Denmark, 5400-4000 BC. (Unpublished Dissertation, Univ. of Wisconsin-Madison, 2010).

35. Hoffman, B. W., Czederpiltz, J. M. & Partlow, M. A. Heads or tails: the zooarchaeology of Aleut salmon storage on Unimak Island, Alaska. *J. Archaeol. Sci.* **27**, 699-708 (2000).

36. Morrison, D. Inuvialuit fishing and the Gutchiak site. *‎Arct. Anthropol.* **37**, 1-42 (2000).

37. Howse, L. Late Dorset caribou hunters: zooarchaeology of the Bell site, Victoria Island. *Arct. Anthropol.***45**, 22-40 (2008).

38. Friesen, M. The Last Supper: Late Dorset Economic Change at Iqaluktuuq, Victoria Island. In *The Northern World AD 900-1400.* (eds Maschner, H., Mason, O. & McGhee, R.) 235-248 (Univ. Utah Press, 2009).

39. Norman, L. & Friesen, M. Thule fishing revisited: the economic importance of fish at the Pembroke and Bell sites, Victoria Island, Nunavut. *Danish Journal of Geography* **110**, 261-278 (2010).

40. Koivisto, S. & Nurminen, K. Go with the flow: stationary wooden fishing structures and the significance of estuary fishing in Subneolithic Finland. *Fennoscandia Archaeologica* **32**, 55-77 (2015).

41. Ukkonen, P. Osteological analysis of the refuse fauna in the Lake Saimaa area. In T. Kirkinen (ed.), *Environmental Studies in Eastern Finland: Reports of the Ancient Lake Saimaa Project: 63-91*. Helsinki Papers in Archaeology 8. University of Helsinki, Helsinki (1996).

42. Mannermaa, K. *The Archaeology of Wings: Birds and People in the Baltic Sea Region during the Stone Age.* Author’s edition, Helsinki (2008).

43. Nurminen, K. Taphonomy of burned fish bones - burning experiments in the open fire. *Environmental Archaeology* **21**, 157-160 (2016).

44. Vilkuna, K. Lohi: Kemijoen ja sen lähialueen lohenkalastuksen historia. Otava, Keuruu (1974).

45. Koivisto, S. Archaeology of Finnish wetlands: with special reference to studies of Stone Age stationary wooden fishing structures. Unigrafia, Helsinki (2017).

46. Halinen, P., Katiskoski, K. & Sarkkinen, M.. Yli-Iin Kuuselankankaan asuinpaikan tutkimukset 1994–1996. In H. Ranta (ed.), *Kentältä poimittua 4: Kirjoitelmia arkeologian alalta*: 24-40. Museoviraston arkeologian osaston julkaisuja No 7. (National Board of Antiquities, Helsinki 1998).

47. Jungner, H. & Sonninen, E. *Radiocarbon dates* 5. (Dating Laboratory, University of Helsinki, 1996).

48. Nordqvist, K. & Mökkönen, T. Periodisation of the Neolithic and radiocarbon chronology of the early Neolithic and the beginning of the Middle Neolithic in Finland. *Documenta Praehistorica* **XLIV**, 78-86 (2017).

49. Online register of archaeological sites and research reports. https://www.kyppi.fi/palveluikkuna/portti/read/asp/default.aspx (National Board of Antiquities, 2018).

50. Jungner, H. & Sonninen, E. *Radiocarbon Dates* 6. (Dating Laboratory, University of Helsinki, 2004).

51. Pesonen, P. *et al*. Early Subneolithic ceramic sequences in eastern Fennoscandia - a Bayesian approach. *Radiocarbon*, **54,** 661-676 (2012).

52. Núñez, M. & Okkonen, J. Humanizing of north Ostrobothnian landscapes during the 4th and 3rd millennia BC. *Journal of Nordic Archaeological Science* **15**, 25-38 (2005).

53. Vaneeckhout, S. Sedentism on the Finnish northwest coast: shoreline reduction and reduced mobility. *Fennoscandia Archaeologica* **25,** 61-72 (2008).

54. Nordqvist, K., Herva, V.-P., Ikäheimo, J. & Lahelma, A. Early copper use in Neolithic northeastern Europe: an overview. *Estonian Journal of Archaeology* **16**, 3-25 (2012).

55. Koivisto, S. Subneolithic fishery in the Iijoki river estuary, northern Ostrobothnia, Finland.

*Journal of Wetland Archaeology* **12**, 22-47 (2012).

56. Butler, D. H. & Shahack-Gross, R. formation of biphasic hydroxylapatite-beta magnesium tricalcium phosphate in heat treated salmonid vertebrae. *Sci. Rep*. **7**, 3610-3621 (2017).

57. Jang, H. L. *et al*. Revisiting whitlockite, the second most abundant biomineral in bone: nanocrystal synthesis in physiologically relevant conditions and biocompatibility evaluation. *ACS nano* **8**, 634-641 (2013).

58. Gende, S. M. *et al*. Pacific salmon in aquatic and terrestrial ecosystems: Pacific salmon subsidize freshwater and terrestrial ecosystems through several pathways, which generates unique management and conservation issues but also provides valuable research opportunities. *BioScience* **52**, 917-928 (2002).

59. Li, X. *et al*. Solubility of Mg-containing β-tricalcium phosphate at 25 C. *Actabiomaterialia* **5**, 508-517 (2009).

60. Dorozhkina, E. I. & Dorozhkin, S. V. Mechanism of the solid-state transformation of a calcium-deficient hydroxyapatite (CDHA) into biphasic calcium phosphate (BCP) at elevated temperatures. *Chem. Mater.* **14**, 4267-4272 (2002).

61. Weiner, S. *Microarchaeology*: *Beyond the Visible Archaeological Record*. (Cambridge University Press, 2010).

62. Wipfli, M. S., Hudson, J. & Caouette, J. Influence of salmon carcasses on stream productivity: response of biofilm and benthic macroinvertebrates in southeastern Alaska, USA. *Can. J. Fish. Aquat. Sci.* **55**, 1503-1511 (1998).

63. Myers, R. A. & Hutchings, J. A. Sea age at maturity for Atlantic salmon, Salmo salar, in North America. *Can. Tech. Rep. Fish. Aquat. Sci*. **1566** (1987).

64. Butler, D. H. & Dawson, P. C. Accessing hunter-gatherer site structures using Fourier transform infrared spectroscopy: applications at a Taltheilei settlement in the Canadian Sub-Arctic. *J. Archaeol. Sci.* **40**, 1731-1742 (2013).

65. Reidsma, F. H. *et al*. Charred bone: physical and chemical changes during laboratory simulated heating under reducing conditions and its relevance for the study of fire use in archaeology. *J. Archaeol. Sci. Rep* **10**, 282-292 (2016).

66. Snoeck, C., Lee-Thorp, J. A. & Schulting, R. J. From bone to ash: compositional and structural changes in burned modern and archaeological bone. *Palaeogeogr. Palaeoclimatol. Palaeoecol.* **416**, 55-68 (2014).

67. Stiner, M. C., Kuhn, S. L., Weiner, S. & Bar-Yosef, O. Differential burning, recrystallization, and fragmentation of archaeological bone*. J. Archaeol. Sci.* **22**, 223–237 (1995).

68. Weiner, S. & Bar-Yosef, O. States of preservation of bones from prehistoric sites in the

near east: a survey. *J. Archaeol. Sci.* **17**, 187-196 (1990).

69. Lebon, M. *et al*. New parameters for the characterization of diagenetic alterations and heat-induced changes of fossil bone mineral using Fourier transform infrared spectrometry. *J. Archaeol. Sci*. **37**, 2265-2276 (2010).

70. Thompson, T. J. *et al*. A new statistical approach for determining the crystallinity of heat-altered bone mineral from FTIR spectra. *J. Archaeol. Sci.* **40**, 416-422 (2013).

71. Beasley, M. M. *et al*. Comparison of the transmission FTIR, ATR, and DRIFT spectra: implications for assessment of bone bioapatite diagenesis. *J. Archaeol. Sci*. **46**, 16-22 (2014).

72. Shahack-Gross, R. *et al*. Evidence for the repeated use of a central hearth at Middle Pleistocene (300 ky ago) Qesem Cave, Israel. *J. Archaeol. Sci.* **44**, 12-21 (2014).

73.Squires, K. E., *et al* The application of histomorphometry and Fourier transform infrared spectroscopy to the analysis of early Anglo-Saxon burned bone. *J. Archaeol. Sci.* **38**, 2399-2409 (2011).

74. Weiner, S. *Microarchaeology*: *Beyond the Visible Archaeological Record*. (Cambridge University Press, 2010).

75. Shipman, P., Foster, G. & Schoeninger, M. Burnt bones and teeth: an experimental study of colour, morphology, crystal structure and shrinkage. *J. Archaeol. Sci.* **11**, 307-325 (1984).

76. Trueman, C. N., Privat, K. & Field, J. Why do crystallinity values fail to predict the extent of diagenetic alteration of bone mineral? *Palaeogeogr. Palaeoclimatol. Palaeoecol.* **266**, 160-167 (2008).

77. Andrews, L. & Wang, X. Infrared spectra of the group 2 metal dihydroxide molecules. *Inorganic Chemistry* **44**, 11-13 (2005)

78. Bellomo, R. V. A methodological approach for identifying archaeological evidence of fire resulting from human activities. *J. Archaeol. Sci.* **20**, 525-553 (1993).

79. Liedgren, L. G. & Ostlund, L. Heat, smoke and fuel consumption in a high mountain Stallo-hut, northern Sweden - experimental burning of fresh birch wood during winter. *J. Archaeol. Sci.* **38**, 903-912 (2011).

80. Gur-Arieh, S. *et al*. Formation processes in Philistine hearths from Tell es-Safi/Gath (Israel): an experimental approach. *J. Field Archaeol.* **37**, 121-131 (2012).

81. Canti, M. G.. Aspects of the chemical and microscopic characteristics of plant ashes found in archaeological soils. *Catena*. **54**, 339-361 (2003).

82. Shahack-Gross, R *et al*. Bat guano and preservation of archaeological remains in cave sites. *J. Archaeol. Sci.* **31**, 1259-1272 (2004).
